# A Short Tandem Repeat-Enriched RNA Assembles a Nuclear Compartment to Control Alternative Splicing and Promote Cell Survival

**DOI:** 10.1016/j.molcel.2018.08.041

**Published:** 2018-11-01

**Authors:** Karen Yap, Svetlana Mukhina, Gen Zhang, Jason S.C. Tan, Hong Sheng Ong, Eugene V. Makeyev

**Affiliations:** 1 Centre for Developmental Neurobiology, King’s College London, London SE1 1UL, UK; 2 School of Biological Sciences, Nanyang Technological University, Singapore 637551, Singapore

**Keywords:** long noncoding RNA, short tandem repeats, RNA-binding protein, PTBP1, nuclear body, perinucleolar compartment, alternative splicing, cell survival, cell transformation, cancer

## Abstract

Functions of many long noncoding RNAs (lncRNAs) depend on their ability to interact with multiple copies of specific RNA-binding proteins (RBPs). Here, we devised a workflow combining bioinformatics and experimental validation steps to systematically identify RNAs capable of multivalent RBP recruitment. This uncovered a number of previously unknown transcripts encoding high-density RBP recognition arrays within genetically normal short tandem repeats. We show that a top-scoring hit in this screen, lncRNA PNCTR, contains hundreds of pyrimidine tract-binding protein (PTBP1)-specific motifs allowing it to sequester a substantial fraction of PTBP1 in a nuclear body called perinucleolar compartment. Importantly, PNCTR is markedly overexpressed in a variety of cancer cells and its downregulation is sufficient to induce programmed cell death at least in part by stimulating PTBP1 splicing regulation activity. This work expands our understanding of the repeat-containing fraction of the human genome and illuminates a novel mechanism driving malignant transformation of cancer cells.

## Highlights

• Human genome encodes many transcripts enriched in short tandem repeats (strRNAs)
• strRNA PNCTR recruits RNA-binding protein PTBP1 to a nuclear body called PNC
• PNCTR antagonizes splicing regulation function of PTBP1 and promotes cell survival
• PNCTR is dramatically upregulated in a wide range of cancer cells

## Graphical Abstract

**Figure:**
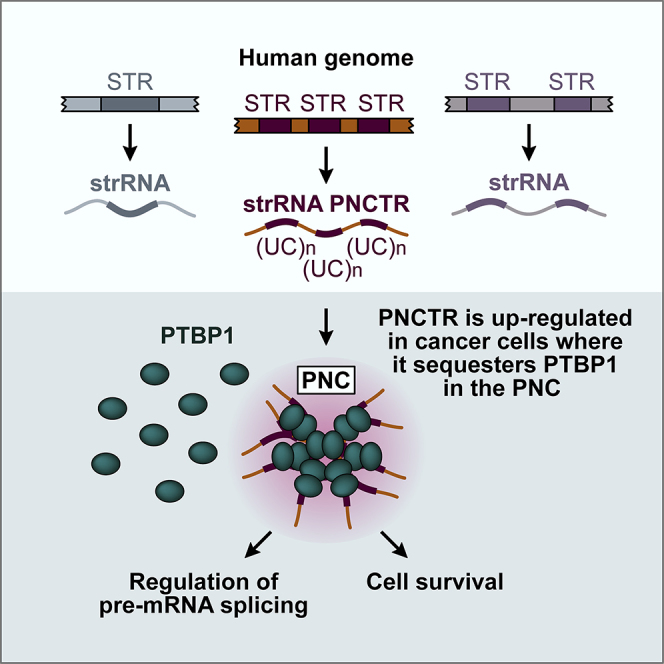

## Teaser

Yap et al. show that several short tandem repeats (STRs) encoded in the human genome are transcribed at a readily detectable level. The STR-enriched RNA PNCTR sequesters multiple copies of PTBP1 in the perinucleolar compartment, thus modulating splicing regulation function of this RNA-binding protein and promoting cancer cell survival.

## Introduction

Functions of many lncRNAs, >200-nt-long transcripts lacking functional open reading frames (ORFs), depend on recruitment of multiple copies of specific RNA-binding proteins (RBPs) to repeated *cis*-elements (Deveson et al., 2017, Quinn and Chang, 2016). For example, a decoy long noncoding RNA (lncRNA) called NORAD contains at least 17 binding sites for the RBP Pumilio (Lee et al., 2016, Tichon et al., 2016). Another lncRNA, Firre, may control nuclear architecture through repeat-mediated interaction with the nuclear matrix protein hnRNP U (Hacisuleyman et al., 2014).

Several lncRNAs function as scaffolds facilitating biogenesis of non-membrane-bound cellular compartments (Chujo and Hirose, 2017, Staněk and Fox, 2017, Sun et al., 2017). A classic example is the ribosome-producing organelle nucleolus that requires transcription of the 47S/45S rRNA precursors (pre-rRNA) by the RNA polymerase I for its assembly (Németh and Grummt, 2018). Other structural lncRNAs include NEAT1/MEN-epsilon/beta nucleating paraspeckles, stress-induced Sat-III transcripts involved in nuclear stress body assembly and Hsr-omega RNAs is required to form omega speckles (Chujo and Hirose, 2017, Staněk and Fox, 2017, Sun et al., 2017). Interestingly, the Sat-III and the Hsr-omega RNAs contain 160- to 280-nt-long tandem repeats that may engage in multivalent interactions with corresponding RBPs (Chujo and Hirose, 2017, Staněk and Fox, 2017).

Perhaps the most compelling example of multivalent recruitment of RBPs to RNA is provided by aberrant transcripts expressed in the context of neurodegenerative and neuromuscular disorders and containing genetically expanded short tandem repeats (STRs), head-to-tail concatemers of 2- to 12-nt sequence units (Goodwin and Swanson, 2014, Morriss and Cooper, 2017). For instance, pre-mRNAs containing expanded (CUG)n and (CCUG)n sequences contribute to pathogenesis of myotonic dystrophy by sequestering the RBP Muscleblind (MBNL1) in nuclear foci and inhibiting its splicing regulation function (Goodwin and Swanson, 2014, Morriss and Cooper, 2017).

STRs occupy >3% of the reference human genome (Ellegren, 2004). However, with a notable exception of the subtelomeric repeat-containing lncRNA TERRA, the overall expression status of endogenously encoded STRs and possible biological functions of the corresponding transcripts remain poorly understood (Azzalin and Lingner, 2015, Biscotti et al., 2015). Moreover, it is likely that STR-containing RNAs are underrepresented in the existing transcriptome annotations because of the inherent difficulty in distinguishing such sequences from their close homologs, especially in the context of RNA sequencing (RNA-seq) experiments.

LncRNAs and RBPs are frequently deregulated in cancer (Pereira et al., 2017, Schmitt and Chang, 2016). For example, polypyrimidine tract-binding protein (PTBP1/PTB/hnRNP I), an RBP regulating pre-mRNA processing in the nucleus and mRNA translation in the cytoplasm (Kafasla et al., 2012, Keppetipola et al., 2012), is upregulated in several types of cancer (Cheung et al., 2009, He et al., 2014, Wang et al., 2017). This has been linked with increased proliferation and invasiveness of cancer cells, as well as their ability to carry out aerobic glycolysis and evade apoptosis induced by extrinsic cues (Cheung et al., 2009, Cobbold et al., 2010, David et al., 2010, Izquierdo et al., 2005, Wang et al., 2017). PTBP1 is also a natural repressor of differentiation-specific alternative splicing events (Boutz et al., 2007, Kafasla et al., 2012, Keppetipola et al., 2012, Makeyev et al., 2007, Spellman et al., 2007, Yap et al., 2012), providing another possible explanation for its increased expression in cancer cells.

However, upregulation of PTBP1 is insufficient to trigger cellular transformation on its own (Wang et al., 2008a). Possibly explaining this paradox, PTBP1 has been shown to stimulate expression of several activators of apoptosis by either altering splicing of their pre-mRNAs or increasing their translation efficiency (Bielli et al., 2014, Bushell et al., 2006, Zhang et al., 2009). How these pro-apoptotic activities are managed in transformed cells overexpressing PTBP1 is an open question. In many cancer cells, a fraction of PTBP1 is recruited to a nuclear body called perinucleolar compartment (PNC) (Ghetti et al., 1992, Matera et al., 1995, Norton and Huang, 2013), but the functional significance of this effect and the mechanisms directing PTBP1 to the PNC remain unclear.

Here, we used a combination of bioinformatics and experimental approaches to uncover a number of previously unknown STR-enriched lncRNAs predicted to recruit multiple copies of cognate RBPs. An in-depth analysis of one such lncRNA reveals its critical roles in PNC assembly, regulation of PTBP1 activity, and cell survival.

## Results

### Systematic Identification of RNAs Capable of Multivalent RBP Recruitment

**Figure 1:**
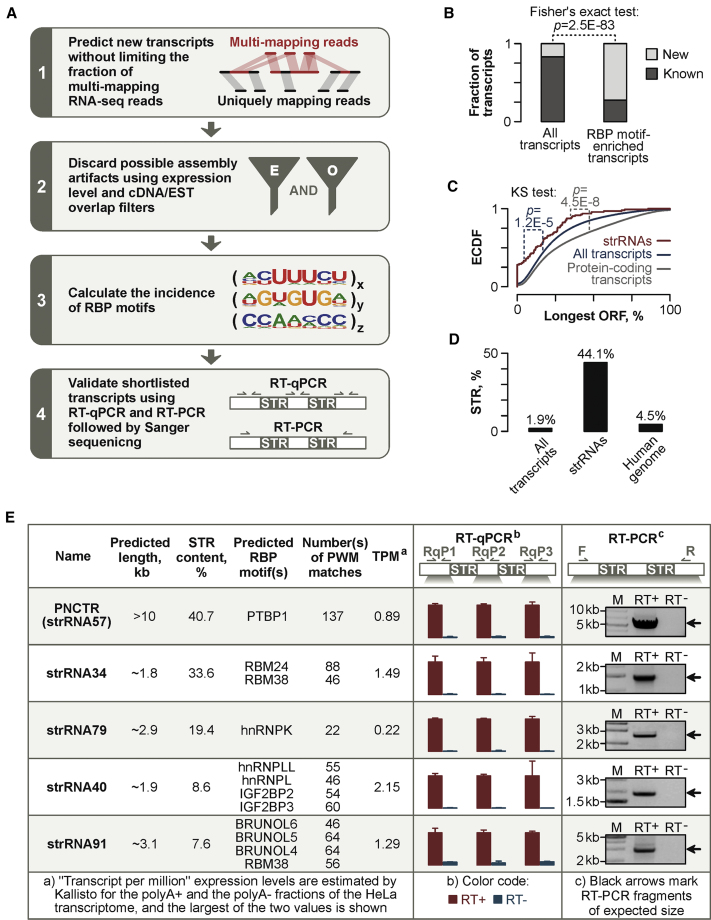
Identification of strRNAs Enriched in RBP Interaction Motifs (A) Workflow used in this study. (B) Transcripts newly predicted by the pipeline in (A) (“new”) are significantly over-represented among RBP motif-enriched RNAs as compared to previously annotated (“known”) transcripts. (C) strRNAs have significantly shorter ORFs compared to annotated mRNAs and the entire transcriptome. (D) STR content of strRNAs substantially exceeds corresponding transcriptome and genome values. (E) qRT-PCR and RT-PCR validation of five newly identified strRNAs using samples prepared without reverse transcriptase (RT) as negative controls. Data are shown as mean ± SD. See also Figure S1 and Table S1.

To predict transcripts that may interact with multiple copies of specific RBPs, we devised a hybrid workflow that reassembles the transcriptome from RNA-seq data without limiting the contribution of multi-mapping reads and enriches true positives through a series of bioinformatics filters and experimental validation steps (Figure 1A; see STAR Methods for more detail). Implementing the first two steps of the workflow for five commonly used human cell lines (A549, HeLa-S3, HepG2, K562, and MCF7) extended the GENCODE annotation by ∼17% of newly predicted transcripts (Figure 1B). Notably, when we examined transcriptome-wide distribution of RBP motifs, the new transcripts were clearly over-represented (Fisher’s exact test p = 2.5 × 10^−83^) among the top hits with *Z* scores for motif number and density ≥5 (Figure 1B).

Of the newly predicted transcripts, 96 were classified as “unknown intergenic RNAs” (StringTie class code “u”; Table S1). These tended to have limited protein-coding capacity (Figure 1C), a feature characteristic for lncRNAs, and an unusually high STR content (44.1%) exceeding the overall transcriptome (1.9%) and genome (4.5%) values (Figure 1D). We therefore termed these transcripts strRNAs. Encouragingly, one strRNA (strRNA64; Table S1) originated from a subtelomeric region, contained TERRA-like (UUAGGG)n repeats, and was predicted by our pipeline to interact with hnRNPA1, a known RBP partner of TERRA (Azzalin and Lingner, 2015). Further searches showed that only four additional strRNAs partially overlapped previously annotated (but not experimentally characterized) lncRNAs (Table S1). To the best of our knowledge, the remaining strRNAs have not been documented previously.

Five strRNAs selected for experimental validation were readily detectable in HeLa cells using qRT-PCR analyses with three primer pairs against the 5′-proximal, middle and 3′-proximal parts of the predicted transcript sequence (Figure 1E). We also successfully amplified large STR-containing fragments of these transcripts using regular RT-PCR and confirmed their identities by Sanger sequencing (Figures 1E and S1). Amplification of genomic DNA in the qRT-PCR experiments was ruled out by including corresponding RT-negative controls (Figure 1E).

Thus, the human genome encodes a number of previously unknown STR-enriched RNAs with a strong RBP-interaction potential.

### PNCTR Is a Long Transcript Produced by RNA Polymerase I

**Figure 2:**
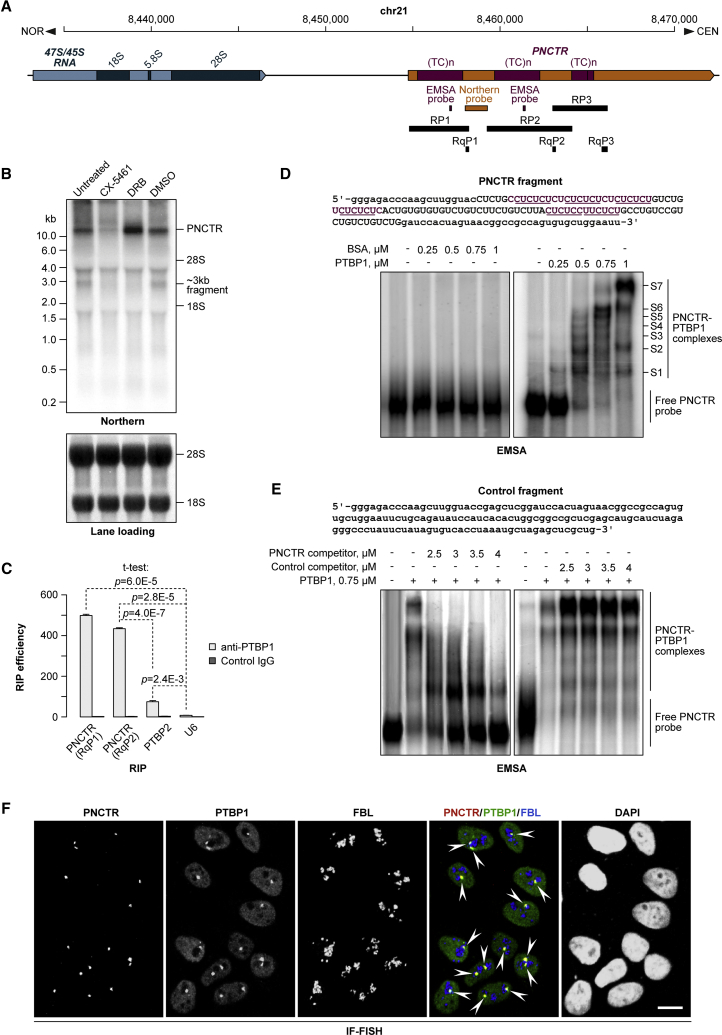
PNCTR Is a pol-I Transcript Interacting with Multiple Copies of PTBP1 Protein (A) Diagram of the predicted *PNCTR* locus also showing an adjacent *47S/45S* rRNA gene and probes used in this study. Mapping to chr21 should be considered provisional since different IGS sequences share extensive regions of homology, and not all parts of human rDNA have been sequenced. (B) Top: northern blot analysis of PNCTR expression in HeLa cells using the probe introduced in (A). Bottom: methylene-blue-stained membrane showing that the lanes were loaded equally. (C) RIP carried out with a PTBP1-specific antibody or a non-immune IgG control. Immunoprecipitated RNAs were analyzed by qRT-PCR using primers specific to PNCTR, PTBP2 pre-mRNA (positive control), or U6 snRNA (negative control). Data are averaged from three experiments ± SD and compared by a two-tailed t test. (D) EMSA with purified PTBP1 protein and a PNCTR-specific RNA probe (sequence on the top). Bottom right: multivalent complexes assemble on incubating the probe with increasing amounts of PTBP1. Bottom left: no band shifts are detected when PTBP1 is substituted with BSA. (E) The PTBP1-PNCTR interaction in (D) is specific since it can be disrupted by increasing amounts of unlabeled PNCTR probe (bottom left), but not a control competitor (top, control RNA sequence; bottom right, the EMSA result). (F) IF-FISH staining of HeLa cells showing that PNCTR co-localizes with PTBP1 in the perinucleolar compartment (PNC). FBL, nucleolar marker fibrillarin. Scale bar, 10 μm. See also Figure S2.

One of the newly identified strRNAs (strRNA57) was encoded in an rDNA intergenic spacer (IGS) and contained numerous PTBP1-specific motifs (Figure 2A). This suggested an alternative name for this transcript: pyrimidine-rich noncoding transcript, or PNCTR. Northern blot analysis with a probe against an STR-depleted part of PNCTR detected >10-kb-long RNA species in HeLa cells (Figures 2A and 2B). An ∼3-kb product was also visible, but it was substantially less abundant (Figure 2B). The probe contained a 186-nt sequence 99% complementary to the IGS28 RNA, an IGS-derived <0.5-kb acidosis-inducible transcript (Audas et al., 2012). However, we failed to detect discrete bands in the corresponding part of the gel suggesting that HeLa cells do not produce substantial amounts of IGS28 under normal conditions (Figure 2B).

Two RNA polymerases, pol I and pol II, can generate PNCTR-sized transcripts. We therefore treated HeLa cells with the pol-I inhibitor CX-5461 or the pol-II inhibitor 5,6-dichloro-1-beta-ribofuranosylbenzimidazole (DRB) and analyzed the samples by qRT-PCR. CX-5461 inhibited the expression of PNCTR, whereas DRB increased its abundance (Figures S2A and S2B). Northern blotting confirmed these results by showing a dramatic decrease in the intensities of the full-length and the ∼3-kb bands in response to CX-5461 (Figure 2B) and accumulation of the full-length band in the DRB-treated sample. DRB also reduced the abundance of the ∼3-kb product, consistent with the possibility that DRB might stabilize PNCTR by a yet-to-be-identified mechanism. As expected for a pol-I transcript, PNCTR did not appear to be capped or polyadenylated (Figures S2C and S2D). Moreover, analysis of publicly available chromatin immunoprecipitation sequencing (ChIP-seq) data confirmed that pol I can form extensive contacts with PNCTR-encoding IGS sequences (Figure S2E).

Overall, this shows that PNCTR occurs predominantly as a >10-kb-long transcript produced by pol I.

### PNCTR Interacts with Multiple Copies of PTBP1 Protein

Predicted PNCTR sequence contains 137 high-quality matches for the PTBP1-specific position weight matrix defined using an *in vitro* selection procedure (Ray et al., 2013) and 2,178 instances of the YUCUYY and the YYUCUY motifs based on *in vivo* patterns of PTBP1 binding (Llorian et al., 2010). Since PTBP1 is a predominantly nuclear protein, we asked whether PNCTR localized in a similar manner. Nuclear and cytoplasmic fractions from control and DRB-treated HeLa cells (Figure S2F) were analyzed by RT-PCR with primers designed to amplify three large STR-containing fragments of PNCTR (RP1–RP3; Figure 2A). The amplification products were readily detectable in the nuclear, but not the cytoplasmic, fraction, and their abundance increased in DRB-treated cells (Figure S2G). All three RT-PCR products had expected lengths arguing against extensive STR expansion in this locus in HeLa cells.

As a direct test for PTBP1/PNCTR interaction, we analyzed HeLa cell lysate by RNA immunoprecipitation (RIP) with a PTBP1-specific antibody. qRT-PCR analysis of the RIP samples showed a robust association of PTBP1 with PNCTR (Figure 2C). PNCTR was immunoprecipitated significantly more efficiently than the PTBP2 pre-mRNA, a previously characterized PTBP1 target (Keppetipola et al., 2012). Confirming specificity of these interactions, the U6 small nuclear RNA (snRNA) that lacks discernable PTBP1 motifs was virtually undetectable in the RIP fraction. Furthermore, PNCTR and PTBP2 pre-mRNA failed to immunoprecipitate when we substituted the PTBP1-specific antibody with a non-immune IgG (Figure 2C).

To further confirm that PTBP1 can interact with PNCTR, we carried out an electrophoretic mobility shift assay (EMSA) with an RNA probe containing ∼0.1-kb PNCTR-derived STR sequence (Figure 2D). Incubation of the probe with increasing amounts of purified recombinant PTBP1 gave rise to several distinct band shifts (S1–S7), whereas no shifts were detected when we substituted PTBP1 with BSA (Figure 2D). Assuming that migration of a complex reflects its PNCTR:PTBP1 stoichiometry, it appears that a single molecule of the probe can interact with ≥6 PTBP1 molecules (Figure 2D), consistent with the presence of 6 non-overlapping and 24 overlapping YUCUYY/YYUCUY motifs in the probe sequence (Figure 2D). The interaction between PTBP1 and PNCTR was specific since the complexes failed to form in the presence of increasing amounts of an unlabeled PNCTR competitor, but not a control RNA lacking PTBP1-specific motifs (Figure 2E).

Thus, PNCTR is a predominantly nuclear RNA capable of recruiting multiple copies of PTBP1.

### PNCTR Localizes to the PNC and Recruits PTBP1 and Possibly Other Proteins to This Nuclear Body

To gain further insights into PNCTR localization, we co-stained HeLa cells with an RNA fluorescence *in situ* hybridization (FISH) probe spanning the entire PNCTR sequence and PTBP1-specific antibody. PNCTR signal typically occurred as one or two prominent dots adjacent to nucleoli (Figure 2F). PTBP1 immunofluorescence (IF) was detectable throughout the nucleoplasm but markedly enriched in perinucleolar foci previously identified as the PNC (Figure 2F; Ghetti et al., 1992, Matera et al., 1995). Strikingly, the PTBP1 foci showed perfect co-localization with the PNCTR dots (Figure 2F). Supporting PNCTR association with the PNC, this strRNA was efficiently immunoprecipitated with an antibody against another PNC marker, CELF1/CUGBP1 (Figure S2H; Norton and Huang, 2013).

To test whether PNCTR could recruit PTBP1 to the PNC, we analyzed cells treated with either CX-5461 or DRB by IF-FISH (Figure S3A). PNCTR dots virtually disappeared in the presence of CX-5461 and became noticeably larger after the addition of DRB (Figure S3A), in line with our biochemical data (Figures 2B, S2A, S2B, and S2G). PTBP1 localization to the PNC was also diminished by CX-5461 and stimulated by DRB (Figure S3A). The PNC marker CELF1 followed a similar trend (Figure S3A). Of note, treating cells with the RNA polymerase-III (pol-III) inhibitor ML-60218 had a relatively mild effect on the PNC morphology (Figure S3B).

**Figure 3:**
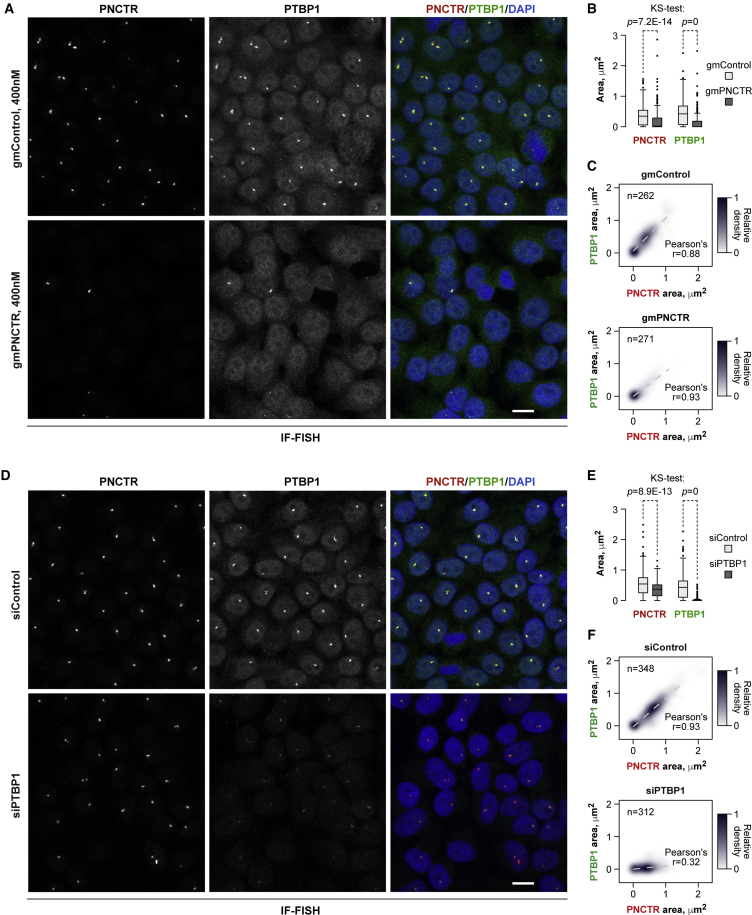
PNCTR Recruits PTBP1 to the PNC (A) HeLa cells were treated for 24 hr with 400 nM gmControl or gmPNCTR and co-stained with a PTBP1-specific antibody and a PNCTR-specific FISH probe. In most nuclei, gmPNCTR eliminates PNC-localized signals in both the PNCTR and PTBP1 channels. (B) Comparison of the dot areas in individual nuclei in (A) using a two-sided Kolmogorov-Smirnov (KS) test. (C) Two-dimensional density plots summarizing the relationship between PNCTR and PTBP1 foci in (A). (D) HeLa cells were incubated with either siControl or siPTBP1 for 48 hr and analyzed by IF-FISH as in (A). Note that siPTBP1 diminishes the size and intensity of both the PTBP1 and the PNCTR signals, but PNCTR is affected to a lesser extent than PTBP1. Scale bars in (A) and (D), 10 μm. (E) Comparison of the dot areas in (D) using a two-sided KS test. (F) Two-dimensional density plots for the relationship between PNCTR and PTBP1 foci in (D). Maximal densities in (C) and (F) were set to 1. See also Figures S3 and S4.

Importantly, both PNCTR and PTBP1 signals localized to the PNC became smaller or disappeared when we knocked down PNCTR using an antisense gapmer oligonucleotide (gmPNCTR) as compared to a non-targeting gapmer (gmControl; Figures 3A, 3B, and S4A–S4C). When used at its most efficient knockdown concentration (400 nM; Figure S4A) gmPNCTR resulted in virtually complete disappearance of the PNCTR and PTBP1 dots (Figure 3A). The PNCTR and the PTBP1 PNC signals correlated strongly in both the gmControl (Pearson’s *r* = 0.88) and the gmPNCTR samples (*r* = 0.93) despite the obvious shift of the latter distribution toward zero (Figure 3C).

As expected, cells treated with a PTBP1-specific siRNA mixture (siPTBP1) had substantially reduced PTBP1 staining in the nucleoplasm and the PNC as compared to a non-targeting siRNA (siControl) (Figures S4D, 3D, and 3E). PNCTR dots also became somewhat smaller in the siPTBP1-treated cells (Figures 3D and 3E). However, this effect failed to match the extent of PTBP1 depletion from the PNC resulting in markedly reduced correlation between the sizes of the PTBP1 and the PNCTR signals in siPTBP1-treated cells (*r* = 0.32) in comparison with siControl (*r* = 0.93) (Figure 3F).

Since PNCTR knockdown also triggered a significant loss of the PNC-localized CELF1 signal (Figures S4E and S4F), we concluded that PTBP1 recruitment to the PNC and likely the overall integrity of this nuclear body depend on PNCTR.

### PNCTR Can Sequester a Substantial Amount of PTBP1

We next wondered what fraction of the total PTBP1 pool could associate with PNCTR. Our qRT-PCR and immunoblot quantifications estimated that a typical HeLa cell contains ∼36 copies of the PNCTR RNA and ∼286,000 copies of the PTBP1 protein l (Figures S5A–S5C). Given that PNCTR encodes 2,178 YUCUYY/YYUCUY sequences and 565 of them are non-overlapping, PNCTR has a capacity to sequester between 27.44% and 7.12% of cellular PTBP1.

**Figure 4:**
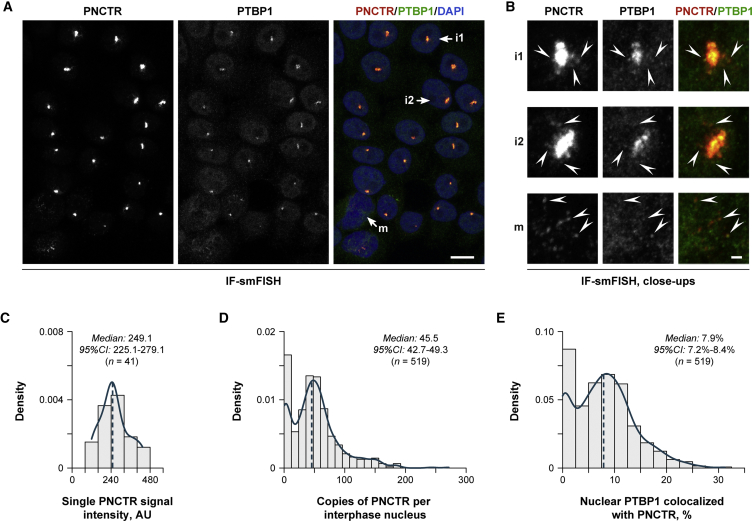
Quantitative Analysis of PTBP1 Sequestration by PNCTR (A) Co-staining untreated HeLa cells with antibodies against PTBP1 and a PNCTR-specific single-molecule FISH (smFISH) probe set confirms that PNCTR and PTBP1 co-localize in the PNC. Scale bar, 10 μm. (B) Inspection of magnified image in (A) additionally reveals individual PNCTR molecules occurring as diffraction-limited spots near the PNC in interphase nuclei (i1 and i2) or distributed throughout DAPI-positive area in cells entering mitosis (m). Arrowheads mark examples of PNCTR molecules co-localizing with PTBP1. Scale bar, 1 μm. (C) Individual PNCTR molecules in (B) give rise to relatively uniform FISH signal intensities. (D) PNCTR abundance calculated as a ratio between the total PNCTR fluorescence per interphase nucleus in (A) and the median intensity of individual PNCTR molecules from (C). (E) Fraction of PTBP1 co-localizing with PNCTR in interphase nuclei in (A). In (C)–(E), solid teal lines show kernel density estimates for the histogram data, and dashed teal lines mark the medians. See also Figure S5.

To further validate this prediction, we co-stained HeLa cells with the anti-PTBP1 antibody and a PNCTR-specific single-molecule RNA FISH probe set (IF-smFISH). The PNC patterns generated in this experiment were virtually indistinguishable from the IF-FISH data above (Figure 4A). However, a closer inspection of magnified images additionally revealed a few diffraction-limited smFISH signals clustered around the PNC in interphase cells or distributed diffusely in cells entering mitosis (Figure 4B). These spots likely corresponded to individual PNCTR molecules because they had relatively uniform size and intensity and often co-localized with PTBP1 IF maxima (Figures 4B and 4C). By dividing a total smFISH signal by the median intensity of individual PNCTR molecules we estimated the median number of PNCTR molecules per interphase nucleus at 45.5 (Figure 4D; 95% confidence interval [CI]: 42.7–49.3), i.e., comparable to the qRT-PCR data in Figure S5A.

Robust detection of both PTBP1 and PNCTR using this protocol also allowed us to estimate the fraction of PTBP1 co-localizing with PNCTR by directly quantifying IF-smFISH images. This suggested that ∼7.9% (median value; 95% CI: 7.2%–8.4%) PTBP1 in HeLa nuclei might interact with PNCTR, and this value reaches 11.4%–31.2% in the upper quartile of the distribution (Figure 4E). Overall, this suggests that a substantial fraction of PTBP1 can occur in a PNCTR-associated form.

### PNCTR Is Required for Cell Survival

**Figure 5:**
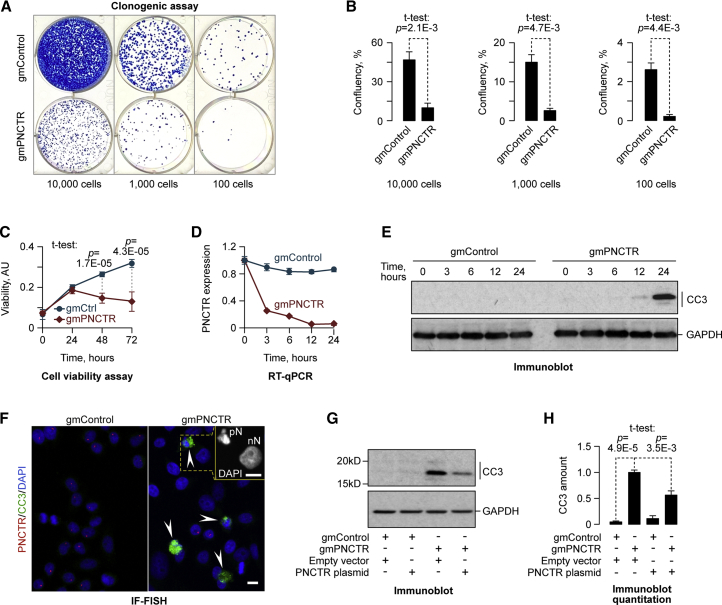
PNCTR Knockdown Promotes Programmed Cell Death (A) HeLa cells were transfected with 400 nM gmControl or gmPNCTR and plated at the densities indicated. Note dramatically reduced numbers of colonies in gmPNCTR-treated wells compared to gmControl. (B) Colony confluency in (A) quantified from 3 independent transfection experiments and shown as mean ± SD. p values are calculated using a two-tailed t test. (C) Growth curves of HeLa cells transfected with 400 nM gmControl or gmPNCTR show that gmPNCTR leads to a visible decline in cell viability between 24 and 72 hpt. Data are averaged from 6 transfection experiments ± SD and compared by a two-tailed t test. (D) Time-resolved qRT-PCR analyses showing that gmPNCTR reaches a maximal downregulation effect by 12 hpt. Data are averaged from 3 experiments ± SD. (E) gmPNCTR, but not gmControl, induces expression of the apoptotic marker cleaved caspase-3 (CC3) at 12–24 hpt. (F) Dampening PNCTR levels often leads to extensive activation of caspase-3 in HeLa cells (arrowheads). The close up in the top-right corner compares DAPI staining for a normal nucleus (nN) of a CC3-negative cell and a pyknotic nucleus (pN) of a cell undergoing apoptosis. Scale bars, 10 μm. (G) CC3 induction by gmPNCTR is less efficient in HeLa cells expressing a gmPNCTR-resistant PNCTR fragment containing (UC)n repeats compared to the corresponding empty vector control. In (E) and (G), GAPDH is used as a lane-loading control. (H) GAPDH-normalized CC3 expression levels in (G) averaged from 3 experiments ± SD and compared by a two-tailed t test. See also Figure S5.

To elucidate biological function of PNCTR, we examined the effect of its knockdown on clonogenic potential of HeLa cells (Figures 5A and 5B). Strikingly, gmPNCTR-treated cultures formed significantly fewer colonies than gmControl-treated ones (Figures 5A and 5B). In a time-resolved cell viability assay, growth of HeLa cultures transfected with gmPNCTR or gmControl was statistically indistinguishable until 24 hr post transfection (hpt) (Figures 5C and S5D). However, gmPNCTR-treated cultures began to lag behind the gmControl-treated ones at 48 and 72 hpt at all three gapmer concentrations tested in this experiment (Figures 5C and S5D). At its most biologically efficient concentration (400 nM; Figure S4A) gmPNCTR reduced the number of viable cells beginning from 24 hpt, while the growth curves of the corresponding gmControl-treated cultures were apparently normal (Figure 5C). Notably, a virtually complete downregulation of PNCTR by gmPNCTR occurred by 12 hpt (Figure 5D), i.e., preceding the viability decline.

To check whether the above effects could be due to programmed cell death, we repeated the gapmer experiment and analyzed expression of a key apoptotic factor, cleaved caspase-3 (CC3), over a 24-hr time period (Figure 5E). A CC3-specific immunoblot signal became detectable in gmPNCTR-treated, but not in gmControl-treated, cultures at 12 hpt, and its intensity further increased by 24 hpt, thus closely matching the PNCTR downregulation kinetics (Figures 5D and 5E). Although the p53 pathway is largely attenuated in HeLa cells, we were able to detect upregulation of this pro-apoptotic tumor suppressor in gmPNCTR-treated, but not gmControl-treated, samples using a sensitive enhanced chemiluminescence protocol (Figure S5E). Our additional IF analyses showed that gmPNCTR led to the appearance of CC3-positive cells often containing pyknotic nuclei, a morphological marker of apoptosis (Figure 5F). Notably, gmPNCTR induced CC3 less efficiently when it was introduced into HeLa cells pre-transfected with an expression plasmid encoding a (UC)n STR-containing PNCTR fragment lacking gmPNCTR-complementary sequences (Figures 5G, 5H, and S5F).

Thus, downregulation of PNCTR triggers apoptosis pointing at its potential pro-survival function.

### PNCTR Antagonizes PTBP1 Splicing Regulation Function

**Figure 6:**
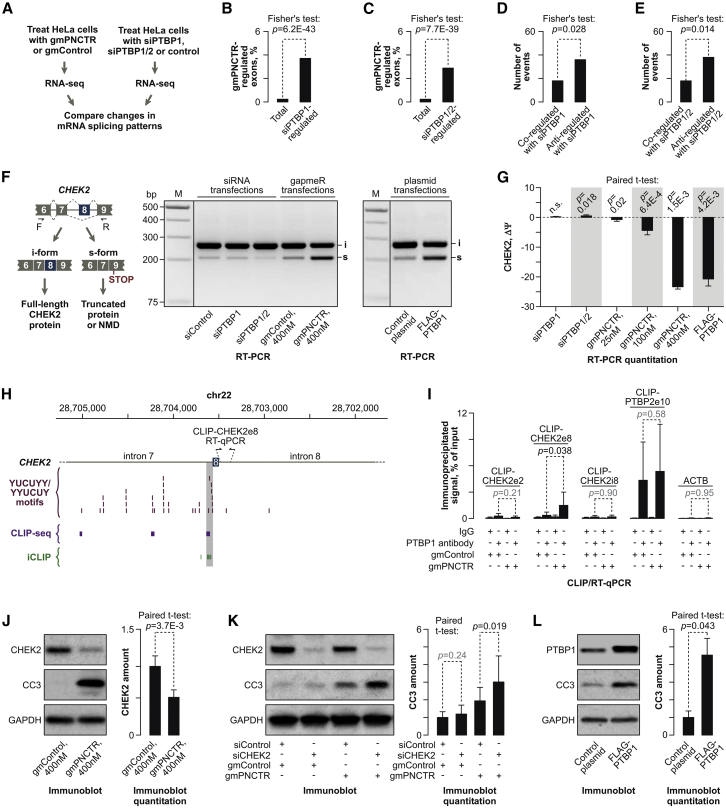
PNCTR Antagonizes Splicing Regulation Function of PTBP1 (A) RNA-seq analyses carried out to examine possible role of PNCTR in modulating PTBP1 activity as a regulator of alternative splicing. (B–E) Fisher’s exact tests showing that gmPNCTR-regulated alternative splicing events are significantly enriched among those regulated by (B) siPTBP1 or (C) siPTBP1/2, as compared to their occurrence in the entire list of alternative splicing events (Total) considered by ExpressionPlot. Note that alternative events controlled by both (D) gmPNCTR and siPTBP1 or (E) gmPNCTR and siPTBP1/2 are preferentially regulated in opposite directions (anti-regulated) rather than the same direction (co-regulated). (F) Regulation of CHEK2 exon 8 splicing by the PNCTR/PTBP1 circuitry. Left: the two alternative splicing possibilities. Right: RT-PCR analyses of HeLa cells showing that combined knockdown of PTBP1 and PTBP2 (siPTBP1/2) stimulates exon 8 inclusion, while knockdown of PNCTR (gmPNCTR, 400 nM) or overexpression of recombinant FLAG-tagged PTBP1 promotes its skipping. (G) Effects in (F) presented as differences in percent-spliced-in values (ΔΨ; Wang et al., 2008b) between experimental treatments and the corresponding controls. Positive ΔΨ values indicate an increase and negative, a decrease in exon 8 inclusion. Similar quantifications were also done for cells transfected with 25 and 100 nM gapmers. All data are averaged from 3 experimentally independent comparisons ± SD and analyzed by a paired t test. (H) CLIP-seq and iCLIP analyses show that PTBP1 forms physical contacts with an extensive array of YUCUYY and YYUCUY motifs in front of CHEK2 exon 8. Functional significance of the PTBP1 interaction sequence highlighted in gray was validated in the minigene experiment in Figures S6L and S6M. (I) CLIP/qRT-PCR experiment showing an increase in PTBP1 interaction efficiency with the CHEK2 exon 8 region (CLIP-CHEK2e8) in HeLa cells treated with gmPNCTR and a lack of this effect for an upstream (CLIP-CHEK2e2) and a downstream region (CLIP-CHEK2i8). PTBP2 pre-mRNA exon 10 region (CLIP-PTBP2e10) and ACTB mRNA are used as a positive and a negative control, respectively. Data are averaged from two triplicated CLIP/qRT-PCR experiments ± SD and compared by a two-tailed t test. (J) Left: immunoblot analysis showing a decrease in the CHEK2 protein levels in HeLa cells transfected for 24 hr with gmPNCTR compared to gmControl. CC3 is used as a sample identity marker and GAPDH as a lane-loading control. Right: immunoblot quantification showing GAPDH-normalized CHEK2 expression levels averaged from 3 experiments ± SD and compared by paired t test. (K) Left: HeLa cells treated with 50 nM of either siControl or siCHEK2 for 36 hr were post-transfected with 400 nM of gmPNCTR or gmControl for 12 hr and analyzed for CC3 expression. Note that the preemptive knockdown of CHEK2 facilitates induction of CC3 in the gmPNCTR samples. Right: GAPDH-normalized CC3 expression averaged from 6 experiments ± SD and compared by paired t test. (L) Expression of recombinant PTBP1 is sufficient to upregulate CC3 in HeLa cells. Left: immunoblot analysis of control and FLAG-PTBP1-transfected samples. Right: GAPDH-normalized CC3 expression averaged from 3 experiments ± SD and compared by paired t test. See also Figures S5 and S6 and Table S2.

We wondered whether the pro-survival activity of PNCTR might depend on its interaction with PTBP1, a known activator of the intrinsic branch of apoptosis. To this end, we analyzed possible changes in HeLa pre-mRNA splicing in response to PNCTR knockdown (Figure 6A). A number of regulated alternative splicing events were indeed detected by comparing gmPNCTR- and gmControl-treated RNA-seq samples using two bioinformatics pipelines, ExpressionPlot (Friedman and Maniatis, 2011) and MISO (Katz et al., 2010). Notably, splicing changes induced by gmPNCTR were enriched among events triggered by treating HeLa cells with siRNAs against PTBP1 (siPTBP1) or both PTBP1 and its functionally similar paralog PTBP2 (siPTBP1/2) (Figures 6B, 6C, and S5G). The fold enrichment of PNCTR-regulated exons among PTBP1-regulated ones increased when we considered more reliably predicted events (Figures S5H and S5I). Importantly, overlapping events regulated in opposite directions (“anti-regulated”) were significantly enriched compared to co-regulated ones (Figures 6D, 6E, and S5J).

We selected five representative examples of anti-regulated events (Table S3) for RT-PCR validation (Figures 6F, 6G, and S6A–S6E). In 4 out of the 5 cases including pre-mRNAs of transcriptional regulators BRD8 and RWDD1, a chloride channel (CLCN6) and a pyruvate carboxylase (PC), knocking down PTBP1 alone (siPTBP1) or in combination with PTBP2 (siPTBP1/2)-stimulated exon inclusion (Figures S6A–S6E). On the other hand, gmPNCTR progressively increased exon skipping in a concentration-dependent manner, and a similar effect was achieved by PTBP1 overexpression (Figures S6A and S6B). gmPNCTR had no detectable effect on the overall PTBP1 protein levels (Figures S6E and S6F) indicating that PNCTR knockdown likely increases PTBP1 activity by changing its cellular localization.

The only example where our routinely used siPTBP1 reagent was ineffective without siPTBP2 was splicing of the cassette exon 8 in the pre-mRNA encoding checkpoint kinase 2 (CHEK2; Figures 6F and 6G). However, switching to a more potent PTBP1-specific siRNA (siPTBP1#7) or overexpressing a (UC)n-containing PNCTR fragment reduced exon 8 skipping in a modest but statistically significant manner (Figures S6G–S6K). Moreover, treating cells with gmPNCTR or overexpressing a FLAG-tagged PTBP1 promoted efficient skipping of this exon (Figures 6F and 6G).

Crosslinking and immunoprecipitation (CLIP)-seq and individual nucleotide resolution CLIP (iCLIP) data available for HeLa cells (Coelho et al., 2015, Haberman et al., 2017, Xue et al., 2009) suggested that the CHEK2 pre-mRNA contains a cluster of functional PTBP1 binding sites in front of exon 8 (Figure 6H). Importantly, qRT-PCR analyses of UV-cross-linked and partially fragmented PTBP1-RNA complexes showed a significant increase in PTBP1 occupancy in the exon 8 region in response to gmPNCTR (Figure 6I; CLIP-CHEK2e8). This was not the case for an upstream and a downstream region of the CHEK2 pre-mRNA depleted for PTBP1-specific CLIP-seq/iCLIP signals (Figure 6I; CLIP-CHEK2e2 and CLIP-CHEK2i8). A strong CLIP/qRT-PCR signal was also detected for a previously described PTBP1 target, PTBP2 pre-mRNA (Figure 6I; CLIP-PTBP2e10). However, this interaction was statistically indistinguishable between the gmControl and the gmPNCTR-treated samples (Figure 6I). Functional importance of the PTBP1-specific motifs preceding CHEK2 exon 8 was further confirmed by our minigene experiments (Figures S6L and S6M).

These data strongly suggest that PNCTR functions as a regulator of splicing antagonizing a specific subset of PTBP1-controlled events.

### PNCTR/PTBP1 Circuitry Controls the Onset and Progression of Apoptosis

CHEK2 regulates important cellular decisions and, depending on circumstances, it can either promote or inhibit apoptosis (Zannini et al., 2014). Since skipping of exon 8 is predicted to truncate CHEK2 protein or/and destabilize its mRNA through nonsense-mediated decay (NMD) (Figure 6F), we hypothesized that the PNCTR/PTBP1 circuitry can control CHEK2 expression levels. Indeed, transfecting HeLa cultures with gmPNCTR for 24 hr decreased CHEK2 protein level ∼2-fold (p = 3.7 × 10^−3^) compared to gmControl (Figure 6J).

To test whether reduced expression of CHEK2 could modulate gmPNCTR-induced apoptotic program, we pre-treated HeLa cells with either CHEK2-specific (siCHEK2) of control siRNAs (siControl) for 36 hr and then transfected the same cultures with gmPNCTR and gmControl for 12 hr, i.e., a time period sufficient for complete downregulation of PNCTR but insufficient for gmPNCTR to activate CC3 to a full extent (Figure 6K). Notably, siCHEK2 significantly stimulated CC3 expression in samples post-transfected with gmPNCTR (Figure 6K) suggesting that downregulation of CHEK2 facilitates gmPNCTR-induced apoptosis.

Although siCHEK2 failed to upregulate CC3 on its own in Figure 6K, PTBP1 is known to alter expression of several other regulators of apoptosis, both at the level of splicing and mRNA translation (Bielli et al., 2014, Bushell et al., 2006, Izquierdo et al., 2005, Zhang et al., 2009). We therefore tested whether increased PTBP1 activity might be sufficient to initiate an apoptotic response in our experimental system. Gratifyingly, transfection of HeLa cells with an expression plasmid encoding a recombinant FLAG-tagged PTBP1 led to a detectable upregulation of CC3 (Figure 6L).

Thus, PNCTR may inhibit apoptosis by limiting cellular PTBP1 activity in general and maintaining adequate expression of CHEK2 in particular.

### PNCTR Is Often Upregulated in Cancer Cells

**Figure 7:**
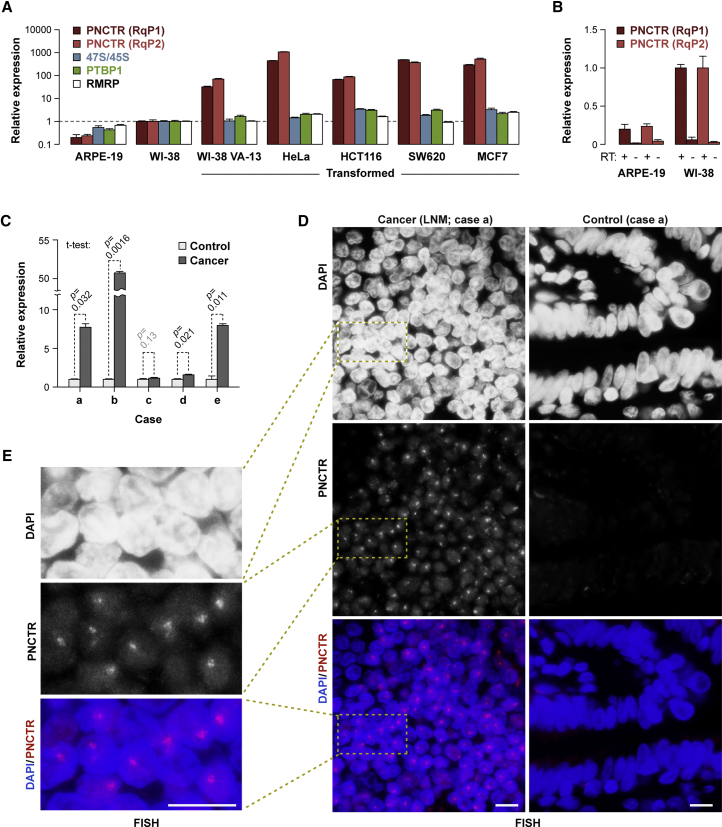
PNCTR Is Often Upregulated in Cancer Cells (A) qRT-PCR analyses showing that PNCTR expression is orders of magnitude higher in transformed cells (HeLa, HCT116, SW620, MCF7, and the SV40-transformed clone VA-13 of the normal lung fibroblast line WI-38) than in their non-transformed counterparts (ARPE-19 and WI-38). The data are averaged from 3 assays ± SD and the expression levels in WI-38 cells were set to 1. (B) qRT-PCR analyses carried out with and without reverse transcriptase (RT) show that the PNCTR signals in ARPE-19 and WI-38 correspond to bona fide expression of this strRNA at low but detectable levels. (C) qRT-PCR comparison of PNCTR expression in five invasive lung cancers and patient-matched normal lung samples (Table S3). Data were obtained using RqP1 primers, normalized to β-actin, averaged from 3 amplification experiments ± SD and compared by a two-tailed t test. (D) PNCTR-positive nuclear dots are readily detectable by RNA-FISH in a lymph node metastasis sample collected for the case (a) in (C), but not in the matching normal lung control. (E) A close up of the boxed area in (D). Scale bars in (D) and (E), 10 μm. See also Figure S7.

Given its pro-survival function we wondered whether PNCTR might be commonly upregulated in transformed cells. Indeed, it was >30-fold more abundant in a SV40-transformed human fibroblast line (WI-38 VA-13) as compared to their non-transformed parental line WI-38 (Figure 7A). A normal epithelial cell line, ARPE-19, expressed somewhat smaller amounts of PNCTR than WI-38 (Figure 7A). On the other hand, steady-state levels of PNCTR in HeLa, colorectal carcinoma HCT116, colorectal adenocarcinoma SW620, and breast adenocarcinoma MCF7 expressed PNCTR were orders of magnitude higher than in WI-38 and ARPE-19 (Figure 7A).

Expression of other RNAs including 47S/45S and RMRP (a pol-III transcript known to localize to the PNC; Matera et al., 1995, Norton and Huang, 2013) did not show an obvious correlation with the cell transformation status (Figure 7A). In line with earlier reports, PTBP1 was noticeably upregulated in all transformed cell lines, but not nearly to the same extent as PNCTR (Figure 7A). The qRT-PCR signals in our assays depended on the presence of RT suggesting that non-transformed cells express PNCTR at low but detectable level (Figure 7B). Cells expressing large amounts of PNCTR also had readily detectable PNCTR- and PTBP1-positive perinucleolar dots (Figures S7A and S7B). Moreover, PNCTR-specific gapmers triggered robust caspase-3 activation in HCT116, but not ARPE-19 (Figures S7C and S7D), consistent with the difference in PNCTR expression between these cells lines (Figure 7A).

To test whether PNCTR was also upregulated in tumor tissues, we analyzed 5 invasive lung cancer biopsies (grade IIB and higher) along with patient-matched normal lung controls. Four out of the 5 tumors expressed PNCTR at a significantly higher level (p < 0.05; t test) than the controls, and in 3 cases PNCTR was upregulated >5-fold (Figure 7C). When we analyzed tissue biopsies available for one of the 5 cases by RNA-FISH, nuclear PNCTR dots were readily detectable in metastatic cancer cells, but not in the normal lung tissue (Figures 7D and 7E).

We finally estimated frequency of cancer-specific PNCTR upregulation by analyzing previously published RNA-seq data for 77 patient-matched pairs of lung cancer and normal lung (Ju et al., 2012). This showed significant upregulation of PNCTR in cancer samples (p = 6.2 × 10^−3^, Wilcoxon signed-rank test; Figure S7E). As a control, we also quantified expression of lncRNA MALAT1 (metastasis-associated lung adenocarcinoma transcript 1) known to be frequently upregulated in cancer (Sun et al., 2017). As expected, MALAT1 levels were significantly higher in cancer samples than in the matching controls (p = 8.0 × 10^−7^, Wilcoxon signed-rank test; Figure S7F). However, PNCTR and MALAT1 were typically upregulated in different patients pointing at different mechanisms underlying these effects (Figure S7F).

We concluded that increased expression of PNCTR is a recurring phenomenon in cancer cells making this strRNA a promising candidate for further biomedical studies.

## Discussion

Our work uncovers previously unknown STR-enriched transcripts (strRNAs) that may function as endogenous regulators of RNA metabolism and subcellular compartmentalization. Similar to the transcripts containing aberrantly expanded STRs and expressed in the context of neurodegenerative and neuromuscular diseases (Goodwin and Swanson, 2014, Morriss and Cooper, 2017), strRNAs are predicted to form multivalent contacts with cognate RBPs. However, an important distinction is that strRNAs contain extensive STR sequences encoded in the reference genome. RT-PCR analyses of five strRNAs expressed at readily detectable levels in HeLa cells are consistent with the lack of repeat expansion at the corresponding loci (Figures 1E and S2G).

We show that one of the strRNAs, PNCTR, recruits multiple copies of PTBP1 protein to the PNC, a cancer-enriched nuclear body (Figures 2, 3, and 4). The PNCTR/CELF1 interaction data (Figures S2H, S3A, S4E, and S4F) further suggest that PNCTR might function as a multipurpose RBP interaction scaffold, similar to several previously characterized lncRNAs (Chujo and Hirose, 2017, Ishizuka et al., 2014, Staněk and Fox, 2017, Wu et al., 2016).

Given that PNCTR is encoded in an rDNA IGS, it is possible that the PNC is nucleated at or near the PNCTR transcription site, in close proximity to the nucleolus assembled at the adjacent genomic sequences. This would explain why HeLa cells typically have one or two PNCs and a larger number of nucleoli (Figure 2F). Similar transcription site-guided process has been proposed for assembly of other nuclear bodies relying on structural lncRNAs, such as paraspeckles, omega speckles, and nuclear stress bodies (Chujo and Hirose, 2017, Staněk and Fox, 2017, Sun et al., 2017). Future studies will show whether this compartmentalization mechanism is more general than currently thought and whether it might involve other newly identified strRNAs.

Another direction for future work will be to understand physicochemical mechanisms underlying PNC assembly. The (CUG)n, (CAG)n and (GGGGCC)n repeats expanded in the context of degenerative diseases can undergo phase transition as a result of intermolecular base-pairing (Jain and Vale, 2017). However, this type of interaction is difficult to envision for PNCTR since (UC)n does not contain pairs of complementary nucleotides. It is more likely that PTBP1, an RBP with four RNA-recognition domains (Kafasla et al., 2012, Keppetipola et al., 2012), functions as a molecular crosslinker in this system. Supporting this possibility, mixtures of PTBP1 and relatively short (UC)n oligonucleotides have been shown to undergo phase separation *in vitro* (Banani et al., 2016). Moreover, PTBP1 knockdown leads to a modest but statistically significant decrease in the size of PNCTR dots (Figures 3D and 3E). The ability of PTBP1 to engage in protein-protein interactions may further enhance its crosslinking properties and explain PNC localization of other components including Raver1/2 and some pol-III transcripts (Kafasla et al., 2012, Keppetipola et al., 2012, Matera et al., 1995, Norton and Huang, 2013).

In addition to its role as a scaffold, PNCTR functions as a decoy molecule sequestering a fraction of cellular PTBP1 in the PNC. This mechanism affects only a subset of PTBP1 targets sensitive to the PTBP1 pool releasable from the PNC depot (Figures 6 and S6). An important example of this behavior is the CHEK2 exon 8 preceded by multiple YUCUYY/YYUCUY motifs that might function as a PTBP1 concentration “sensor” (Figures 6F–6J and S6G–S6M).

Exon 8-dependent changes in CHEK2 protein levels appear to stimulate the apoptotic program triggered by PNCTR knockdown (Figures 5 and 6). This might be further facilitated by the PTBP1-dependent pro-apoptotic events described earlier (Bielli et al., 2014, Bushell et al., 2006, Zhang et al., 2009). Yet another PNCTR/PTBP1 target uncovered in our study, BRD8, encodes a component of the Tip60/NuA4 complex known to control cell survival (Doyon and Côté, 2004). Thus, PNCTR may act as a buffer allowing cancer cells to express sufficiently large amounts of PTBP1 while mitigating the risk of apoptosis. Since PNCTR knockdown tends to promote stronger caspase-3 activation than PTBP1 overexpression (e.g., cf. CC3 upregulation in Figures 6J and 6L), it will be interesting to see whether this strRNA employs additional, PTBP1-independent mechanisms to promote cell survival.

The rDNA region encoding PNCTR is also known to give rise to substantially shorter (<0.5 kb) stress-induced IGS RNAs mediating protein detention in nucleolus (Audas et al., 2012). Although one of the best-characterized examples of this class of transcripts, the acidosis-inducible IGS28 RNA, was not detectable under our experimental conditions (Figure 2B), it is possible that PNCTR is post-transcriptionally processed to generate other short RNAs transported to the nucleolus. This exciting possibility will be explored in the future, along with the role that cell-specific differences in PNCTR turnover might play in regulating its steady-state expression levels. Of note, PNCTR is relatively short lived even in cancer cells (Figure 5D) suggesting that the corresponding IGS locus is actively transcribed by pol I (Figure S2E).

We finally show that PNCTR is upregulated to a remarkable extent in a wide range of human cancers (Figures 7 and S7) explaining the widespread occurrence of PNC in malignant tumors (Norton and Huang, 2013). Perhaps more importantly, this argues that further work on PNCTR and other strRNAs may provide novel insights into disease mechanisms and lead to new therapies and diagnostic approaches.

## STAR★Methods

### Key Resources Table

**Table:** 

REAGENT or RESOURCE	SOURCE	IDENTIFIER
**Antibodies**		

Mouse anti-PTBP1 (1)	Thermo Fisher Scientific	Cat# 32-4800; RRID: AB_2533082
Rabbit anti-CC3 (Asp175)	Cell Signaling Technology	Cat# 9661; RRID: AB_2069869
Mouse anti-GAPDH (6C5)	Thermo Fisher Scientific	Cat# AM4300; RRID: AB_437392
Rabbit anti-Fibrillarin	Abcam	Cat# ab5821; RRID: AB_2105785
Rabbit anti-CUGBP1(3B1)	Millipore	Cat# 05-621; RRID: AB_11211990
Rabbit anti-p44/42 MAPK (Erk1/2)	Cell Signaling Technology	Cat# 9102; RRID: AB_330744
Mouse IgG control (NCG01)	Thermo Fisher Scientific	Cat# MA5-14453; RRID: AB_10943239
IRDye 800CW goat anti-mouse IgG (H+L)	Li-COR Biosciences	Cat# 925-32210; RRID: AB_2687825
IRDye 680RD goat anti-rabbit IgG (H+L)	Li-COR Biosciences	Cat# 926-68071; RRID: AB_10956166
Alexa Fluor 488-conjugated goat anti-mouse IgG (H+L)	Thermo Fisher Scientific	Cat# A-11001; RRID: AB_2534069
Alexa Fluor 647-conjugated streptavidin	Thermo Fisher Scientific	Cat# S21374; RRID: AB_2336066
HRP-conjugated goat anti-rabbit IgG (H+L)	Jackson ImmunoResearch Laboratories	Cat# 111-035-144; RRID: AB_2307391
HRP-conjugated goat anti-mouse IgG (H+L)	Jackson ImmunoResearch Laboratories	Cat# 115-035-146; RRID: AB_2307392

**Bacterial and Virus Strains**		

TOP10	Thermo Fisher Scientific	Cat# C404010
Stbl3	Thermo Fisher Scientific	Cat# C737303
EL250	(Lee et al., 2001)	N/A

**Biological Samples**		

Total RNA samples and formalin fixed paraffin-embedded tissue blocks containing lung metastatic tumors and patient-matched normal lung biopsies	OriGene	See Table S3 for more detail

**Chemicals, Peptides, and Recombinant Proteins**		

5,6-dichloro-1-d-ribofuranosylbenzimidazole (DRB)	Cayman Chemical	Cat# 10010302; CAS# 53-85-0
CX-5461	Cayman Chemical	Cat# 18392; CAS# 1138549-36-6
InSolution RNA Polymerase III Inhibitor	Merck	Cat# 557404 CAS# 577784-91-9
Dimethyl sulfoxide (DMSO)	Sigma Aldrich	Cat# D2650
Phenylmethanesulfonyl Fluoride (PMSF)	New England Biolabs	Cat# 8553S
Recombinant PTBP1	ORFeome	N/A
Lipofectamine 2000 reagent	Thermo Fisher Scientific	Cat# 11668027
Lipofectamine RNAimax reagent	Thermo Fisher Scientific	Cat# 13778075
TRIzol reagent	Thermo Fisher Scientific	Cat# 15596026
RNase inhibitor, murine	New England Biolabs	Cat# M0314
RNasin Ribonuclease Inhibitors (recombinant)	Promega	Cat# N2111
TURBO DNase	Thermo Fisher Scientific	Cat# AM2238
PureLink DNase	Thermo Fisher Scientific	Cat# 12185010
RQ1 DNase	Promega	Cat# M6101
T7 RNA polymerase	Promega	Cat# P2075
T7 RNA polymerase	New England Biolabs	Cat# M0251S
rNTPs	New England Biolabs	Cat# N0450S
Ribo m7G Cap analog	Promega	Cat# P1711
Biotin-16-dUTP	Sigma-Aldrich	Cat# 11093070910
[α-32P] UTP	Perkin Elmer	Cat# NEG007X250UC
[α-32P] dCTP	Perkin Elmer	Cat# NEG513H500UC
SuperScript IV Reverse Transcriptase (RT)	Thermo Fisher Scientific	Cat# 18090010
T4 Polynucleotide Kinase	New England Biolabs	Cat# M0201S
Dynabead Protein G for Immunoprecipitation	Thermo Fisher Scientific	Cat# 10003D
Poly-D-lysine hydrobromide	Sigma-Aldrich	Cat# P7280
MagJet Enrichment kit	Thermo Fisher Scientific	Cat# K2811
RNase I	Thermo Fisher Scientific	Cat# AM2295
Sodium periodate (NaIO_4_)	Sigma Aldrich	Cat# 71859
Biocytin hydrazide	AAT Bioquest	Cat# 3086-AAT
Hydrophilic streptavidin magnetic beads	New England Biolabs	Cat# S1421S
Doxycycline	Sigma Aldrich	Cat# D9891
DAPI	Thermo Fisher Scientific	Cat# D1306

**Critical Commercial Assays**		

PrestoBlue Cell Viability Reagent	Thermo Fisher Scientific	Cat# A13261
Pierce BCA Protein Assay Kit	Thermo Fisher Scientific	Cat# 23227
mMESSAGE mMACHINE T7 Kit	Thermo Fisher Scientific	Cat# AM1344
Amersham Megaprime DNA Labeling Systems	GE Healthcare	Cat# RPN1606
Nick Translation Mix	Sigma-Aldrich	Cat# 11745808910
PureLink RNA Mini kit	Thermo Fisher Scientific	Cat# 12183018A
QIAprep Spin Miniprep Kit	QIAGEN	Cat# 27106
NucleoSpin Gel and PCR Clean-up kit	Macherey-Nagel	Cat# 740609.250

**Deposited Data**		

CSHL_RnaSeq_A549_cell_longNonPolyA	ENCODE; Nature. 2012 Sep 6;489(7414):101-8.	GEO: GSM767854
CSHL_RnaSeq_A549_cell_longPolyA	ENCODE; Nature. 2012 Sep 6;489(7414):101-8.	GEO: GSM758564
CSHL_RnaSeq_HeLa-S3_cell_longNonPolyA	ENCODE; Nature. 2012 Sep 6;489(7414):101-8.	GEO: GSM767847
CSHL_RnaSeq_HeLa-S3_cell_longPolyA	ENCODE; Nature. 2012 Sep 6;489(7414):101-8.	GEO: GSM765402
CSHL_RnaSeq_HeLa-S3_cytosol_longNonPolyA	ENCODE; Nature. 2012 Sep 6;489(7414):101-8.	GEO: GSM767838
CSHL_RnaSeq_HeLa-S3_cytosol_longPolyA	ENCODE; Nature. 2012 Sep 6;489(7414):101-8.	GEO: GSM765404
CSHL_RnaSeq_HeLa-S3_nucleus_longNonPolyA	ENCODE; Nature. 2012 Sep 6;489(7414):101-8.	GEO: GSM767848
CSHL_RnaSeq_HeLa-S3_nucleus_longPolyA	ENCODE; Nature. 2012 Sep 6;489(7414):101-8.	GEO: GSM765403
CSHL_RnaSeq_HepG2_cell_longNonPolyA	ENCODE; Nature. 2012 Sep 6;489(7414):101-8.	GEO: GSM758567
CSHL_RnaSeq_HepG2_cell_longPolyA	ENCODE; Nature. 2012 Sep 6;489(7414):101-8.	GEO: GSM758575
CSHL_RnaSeq_HepG2_cytosol_longNonPolyA	ENCODE; Nature. 2012 Sep 6;489(7414):101-8.	GEO: GSM767840
CSHL_RnaSeq_HepG2_cytosol_longPolyA	ENCODE; Nature. 2012 Sep 6;489(7414):101-8.	GEO: GSM758576
CSHL_RnaSeq_HepG2_nucleus_longNonPolyA	ENCODE; Nature. 2012 Sep 6;489(7414):101-8.	GEO: GSM767850
CSHL_RnaSeq_HepG2_nucleus_longPolyA	ENCODE; Nature. 2012 Sep 6;489(7414):101-8.	GEO: GSM758568
CSHL_RnaSeq_K562_cell_longNonPolyA	ENCODE; Nature. 2012 Sep 6;489(7414):101-8.	GEO: GSM758577
CSHL_RnaSeq_K562_cell_longPolyA	ENCODE; Nature. 2012 Sep 6;489(7414):101-8.	GEO: GSM765405
CSHL_RnaSeq_K562_cytosol_longNonPolyA	ENCODE; Nature. 2012 Sep 6;489(7414):101-8.	GEO: GSM767849
CSHL_RnaSeq_K562_cytosol_longPolyA	ENCODE; Nature. 2012 Sep 6;489(7414):101-8.	GEO: GSM840137
CSHL_RnaSeq_K562_nucleus_longNonPolyA	ENCODE; Nature. 2012 Sep 6;489(7414):101-8.	GEO: GSM767844
CSHL_RnaSeq_K562_nucleus_longPolyA	ENCODE; Nature. 2012 Sep 6;489(7414):101-8.	GEO: GSM765387
CSHL_RnaSeq_MCF-7_cell_longNonPolyA	ENCODE; Nature. 2012 Sep 6;489(7414):101-8.	GEO: GSM767851
CSHL_RnaSeq_MCF-7_cell_longPolyA	ENCODE; Nature. 2012 Sep 6;489(7414):101-8.	GEO: GSM765388
RNA-seq analysis of control-treated HeLa cells	(Ling et al., 2016)	SRA: SRX154426
RNA-seq analysis of HeLa cells with PTBP1 single knockdown	(Ling et al., 2016)	SRA: SRX1544260
RNA-seq analysis of HeLa cells with PTBP1 and PTBP2 double knockdown	(Ling et al., 2016)	SRA: SRX1544257
RNA-seq analyses of primary lung cancer samples and patient-matched controls	(Ju et al., 2012)	ENA: PRJEB2784
Pol I (POLR1A) ChIP-seq in immortalized HMEC cells	(Sanij et al., 2015)	GEO: GSM1544525
PTBP1 CLIP-seq in HeLa cells	(Xue et al., 2009)	GEO: GSE19323
PTBP1 iCLIP in HeLa cells	(Coelho et al., 2015, Haberman et al., 2017)	ArrayExpress: E-MTAB-3108
RNA-seq comparison of HeLa cells treated with control or PNCTR-specific gapmers	This study	ArrayExpress: E-MTAB-6529

**Experimental Models: Cell Lines**		

Human: HeLa	ATCC	Cat# CCL-2; RRID: CVCL_0030
Human: MCF7	ATCC	Cat# HTB-22; RRID: CVCL_0031
Human: SW620	ATCC	Cat# CCL-227; RRID: CVCL_0547
Human: HCT-116	ATCC	Cat# CCL-247; RRID: CVCL_0291
Human: A-549	ATCC	Cat# CCL-185; RRID: CVCL_0023
Human: WI-38	ATCC	Cat# CCL-75; RRID: CVCL_0579
Human: WI-38 VA13 (subline 2RA)	ATCC	Cat# CCL-75.1; RRID: CVCL_2759
Human: ARPE-19	ATCC	Cat# CRL-2302; RRID: CVCL_0145

**Oligonucleotides**		

Negative control A gapmer (gmControl; 5′-A^∗^A^∗^C^∗^A^∗^C^∗^G^∗^T^∗^C^∗^T^∗^A^∗^T^∗^A^∗^C^∗^G^∗^C)	QIAGEN	Cat# 339516 LG00000002-DFA
PNCTR-specific gapmer (gmPNCTR; design ID: LG00170744; 5′-T^∗^G^∗^A^∗^A^∗^G^∗^T^∗^C^∗^G^∗^A^∗^G^∗^G^∗^A^∗^G^∗^C^∗^T^∗^T)	QIAGEN	Cat# 339512 LG00170744-DFA
PNCTR-specific gapmer (gmPNCTR’; design ID:LG00201955; 5′-G^∗^A^∗^C^∗^T^∗^G^∗^T^∗^G^∗^A^∗^C^∗^A^∗^T^∗^A^∗^G^∗^G^∗^T^∗^A	QIAGEN	Cat#339512 LG00201955-DFA
ON-TARGET plus non-targeting siRNA (siControl)	Dharmacon	Cat# D-001810-01-20
Human PTBP1-specific ON-TARGET plus siRNA (siPTBP1#6)	Dharmacon	Cat# J-003528-06
Human PTBP1-specific ON-TARGET plus siRNA (siPTBP1#7)	Dharmacon	Cat# J-003528-07
Human PTBP1-specific ON-TARGET plus siRNA (siPTBP1#8)	Dharmacon	Cat# J-003528-08
Human PTBP1-specific ON-TARGET plus siRNA (siPTBP1#9)	Dharmacon	Cat# J-003528-09
Human PTBP1-specific ON-TARGET plus siRNA SMARTpool (siPTBP1)	Dharmacon	Cat# L-003528-00-0005
Human PTBP2-specific ON-TARGET plus siRNA SMARTpool (siPTBP2)	Dharmacon	Cat# L-021323-01-0005
Human CHEK2-specific ON-TARGET plus siRNA SMARTpool (siCHEK2)	Dharmacon	Cat# L-003256-00-0005
Assorted DNA oligonucleotides	This study/IDT	See Table S5

**Recombinant DNA**		

BAC: CTD-2016H21	Thermo Fisher Scientific	N/A
BAC: PNCTR FISH probe	This study	See Table S4
Plasmid: pcDNA3	Thermo Fisher Scientific	N/A
Plasmid: pEGFP-N3	Clontech	N/A
Plasmid: pEM1032 (Expression plasmid encoding Flag-PTBP1 and EGFP)	(Yap et al., 2012)	N/A
Plasmid: pEM1033 (EGFP control for pEM1032)	(Yap et al., 2012)	N/A
Plasmid: pEM1380 (probe for EMSA)	This study	See Table S4
Plasmid: pML154 (T7-PNCTR)	This study	See Table S4
Plasmid: pML159 (PNCTR fragment expression plasmid containing pol-I promoter)	This study	See Table S4
Plasmid: pBM03 (control vector for pML159)	(Grob et al., 2014)	N/A
Plasmid: pML287 (WT CHEK2 minigene)	This study	See Table S4
Plasmid: pML291 (mut1 CHEK2 minigene)	This study	See Table S4
Plasmid: pML292 (mut2 CHEK2 minigene)	This study	See Table S4

**Software and Algorithms**		

Bowtie2 (Version 2.2.6)	(Langmead and Salzberg, 2012)	http://bowtie-bio.sourceforge.net/bowtie2/index.shtml
TopHat2 (Version 2.1.0)	(Kim et al., 2013)	https://ccb.jhu.edu/software/tophat/index.shtml
HISAT2 (Version 2.1.0)	(Kim et al., 2015)	http://ccb.jhu.edu/software/hisat2/index.shtml
StringTie (Version 1.3.3b)	(Pertea et al., 2015)	https://ccb.jhu.edu/software/stringtie/
Kallisto (Version 0.43.0)	(Bray et al., 2016)	https://pachterlab.github.io/kallisto/about
Bedtools (Version 2.25.0)	(Quinlan and Hall, 2010)	https://bedtools.readthedocs.io/en/latest/
Samtools (Version 1.6)	(Li et al., 2009)	http://www.htslib.org/
FIMO (MEME suite) (Version 4.10.2)	(Bailey et al., 2009, Grant et al., 2011)	http://meme-suite.org/doc/fimo.html
TransDecoder (Version 2.0.1)	(Haas et al., 2013)	https://github.com/TransDecoder/TransDecoder/wiki
ExpressionPlot (Version 0.7)	(Friedman and Maniatis, 2011)	http://www.expressionplot.com/wiki/index.php?title=Main_Page
MISO (Version 0.5.4)	(Katz et al., 2010)	https://miso.readthedocs.io/en/fastmiso/index.html
IGV (Version 2.3)	(Robinson et al., 2011)	https://software.broadinstitute.org/software/igv/download
R (Version 3.4.3)	(R Development Core Team, 2018)	https://www.r-project.org/
ImageJ (Version 1.50c)	NIH	https://imagej.nih.gov/ij/
ImageQuant (Version 5.2)	GE Healthcare Life Sciences	N/A
Image Studio Lite (Version 5.2)	LI-COR Biosciences	N/A
LightCycler 96 software (Version 1.1.0.1320)	Roche	N/A

### Contact for Reagent and Resource Sharing

Further information and requests for reagents may be directed to and will be fulfilled by the Lead Contact, Eugene Makeyev (eugene.makeyev@kcl.ac.uk).

### Experimental Model and Subject Details

#### Cell lines

Human cell lines were maintained in a humidified incubator at 37°C, 5% CO_2_. HeLa, HCT116, SW620 and MCF7 lines were cultured in Dulbecco’s Modified Eagle Medium (DMEM) with high glucose, GlutaMAX and sodium pyruvate (Thermo Fisher Scientific; cat# 31966021) supplemented with 10% Fetal Bovine Serum (FBS; Thermo Fisher Scientific; cat# HYC85), 100 units/ml penicillin and 100 μg/ml streptomycin (1 × PenStrep; Thermo Fisher Scientific; cat# 15140122). ARPE-19, WI-38 and WI-38 VA13 cells were maintained in advanced DMEM/F12 (Thermo Fisher Scientific; cat# 12634010) supplemented with 5% FBS, 4 mM L-glutamine and 1 × PenStrep. In some experiments, cells were treated with either CX5461 (300 ng/mL) or 5,6-dichloro-1-d-ribofuranosylbenzimidazole (DRB; 25 μg/mL) for 5-6 hours. Control samples were treated with DMSO. In transfection experiments, cells were typically seeded overnight in 1 mL of antibiotic-free medium at 2 × 10^5^ per well of a 12-well plate. Next morning, 1 μg plasmid was mixed with 3 μl of Lipofectamine 2000 pre-diluted in 100 μl of Opti-MEM I (Thermo Fisher Scientific; cat# 31985070) and added drop-wise to the cells. Medium was replaced with PenStrep-containing medium 4 hours post transfection and cells were incubated for further 20-68 hours (i.e., 24-72 hours post transfection), as required. To activate expression of doxycycline-inducible TRE promoters in pEM1032 (FLAG-PTBP1), pEM1033 (control plasmid), pML287 (WT CHEK2 minigene), pML291 (mut1 CHEK2 minigene) and pML292 (mut2 CHEK2 minigene), PenStrep-containing medium was additionally supplemented with 2 μg/ml doxycycline (Sigma Aldrich, cat# D9891). In some experiments, transfected cells were enriched by fluorescence activated cell sorting (FACSAria; BD Biosciences) based on the expression of an EGFP marker encoded in the experimental plasmid itself (pEM1032 and pEM1033; Figure 6F) or co-transfected in the form of pEGFP-N3 (Figure S6J). To transfect cells with small nucleic acids, 25-400 pmol of an appropriate gapmer or 50 pmol of an siRNA was mixed with 3 μl of Lipofectamine RNAiMAX pre-diluted in 100 μl of Opti-MEM I (Thermo Fisher Scientific) and incubated with cultures for 24 −72 hours without changing the medium.

### Method Details

#### DNA constructs

Plasmid pBM03 containing a pol-I promoter was kindly provided by Brian McStay (Grob et al., 2014). pcDNA3 and BAC CTD-2016H21 were from Invitrogen/Thermo Fisher Scientific and pEGFP-N3 was from Clontech. The PNCTR FISH probe BAC was derived from CTD-2016H21 by replacing a large 47S/45S-containing fragment with an ampicillin resistance cassette derived from pEM791 (Yap et al., 2012) using recombineering (Lee et al., 2001) (also see Table S4). Other constructs were generated as outlined in Table S4 using routine molecular cloning techniques and enzymes from New England Biolabs. Maps of all constructs are available on request.

#### Nucleocytoplasmic fractionation

Nuclear and cytoplasmic fractions were prepared as described previously (Rio et al., 2010). Briefly, HeLa cells were washed with 1 × PBS, scraped off and centrifuged at 1500 × g, 4°C for 5 min. Cell pellets were resuspended in 4 volumes (relative to the pellet size) of cell disruption buffer (20 mM Tris-HCl pH 7.5, 10 mM KCl, 1.5 mM MgCl_2_ and 1 mM DTT) and incubated for 10 min on ice. This was followed by adding an aliquot of 10% Triton X-100 (Sigma-Aldrich; cat# T8787) to the final concentration of 0.1% and centrifugation at 1500 × g, 4°C for 5 min to separate the cytoplasmic (supernatant) and the nuclear fraction (pellet).

#### DNA probes

To generate a Northern blot probe, PNCTR-specific PCR product amplified with KAPA HiFi polymerase and PNCTR_F5/PNCTR_R5 primers (Table S5) was labeled using [α-32P]-dCTP and Amersham Megaprime DNA Labeling Systems and purified using a G-50 Column (GE Healthcare; cat# 27533001), as recommended. The probe was denatured at 100°C for 5 min and chilled on ice for 2 min immediately before use. Biotin-labeled FISH probe was prepared by incubating 1 μg of the PNCTR FISH probe BAC with 4 μl of 5 × Nick Translation reaction buffer, 0.4 μl of 6 mM dNTP mix, 6 μl 1 mM Biotin-16-dUTP in total volume of 20 μl at 18°C for 2.5 hours. The reaction was stopped by adding 1 μl each of 20% SDS and 0.5 M EDTA followed by a 10-min incubation at 70°C. Labeled DNA fragments were precipitated with 2.5 volumes of ethanol and 0.1 volumes of 3 M sodium acetate (pH 5.2) using 10 μg of salmon sperm DNA (Agilent Technologies; cat# 201190) as a carrier. DNA pellet was washed with 70% ethanol and dissolved in nuclease-free water (Thermo Fisher Scientific, cat# AM9939).

#### RNA probes

Radiolabeled PNCTR RNA fragments used in electrophoretic mobility shift assays (EMSA) were generated by *in vitro* transcription with T7 RNA polymerase (Promega; cat#P2075), as recommended. Briefly, 40 μL reaction mixtures containing 1 × transcription buffer, 10 mM DTT, 0.8 unit/μl rRNasin (Promega; cat#N2111), 0.5 mM ATP, 0.5 mM CTP, 0.2 mM GTP, 0.02 mM UTP, 40 μCi of [α-^32^P]-UTP, 0.8 mM Ribo m7G Cap analog, 0.8 unit/μl T7 RNA polymerase and 1-2 μg of pEM1380 plasmid linearized with EcoRI were incubated for 1 h at 37°C. Unlabeled probes used in EMSA competition assays were produced by transcribing pEM1380 cut with EcoRI (PNCTR fragment) or a PCR fragment amplified from pcDNA3 using KAPA HiFi polymerase (Kapa Biosystems; cat#KK2102) and EMSA_control_F/EMSA_control_R primers (Table S5; control fragment) using the mMESSAGE mMACHINE T7 RNA polymerase kit. In this case, 20 μL reactions containing 1 × reaction buffer, 7.5 mM each of UTP, ATP and CTP, 1.5 mM GTP, 6 mM of the cap analog, 1 μL of the T7 enzyme mix and 1-2 μg of an appropriate DNA template were incubated for 3 h at 37°C. Both radiolabeled- and unlabeled reactions were treated with 2 units of RQ1 DNase (Promega; cat#M6101) for 15 min at 37°C, extracted with acidic phenol-chloroform mixture (1:1), precipitated with 100% ethanol and 3 M sodium acetate, washed with 70% ethanol and re-suspended with DEPC-treated water. To obtain PNCTR RNA fragment used to prepare a standard curve for qRT-PCR quantification, 20 μl reactions containing 1 × RNAPol reaction buffer, 0.5 mM NTP mix, 1 unit of murine RNase inhibitor (New England Biolabs; cat# M0314), 5 mM DTT (Thermo Fisher Scientific; cat# 15508013), 100 units of T7 RNA polymerase (New England Biolabs; cat#M0251S) and 1 μg of pML154 linearized by EcoRI were incubated for 2 hours at 37°C. The RNA was then treated with 2 units of Turbo DNase (Thermo Fisher Scientific; cat#AM2238) for 15 min at 37°C and precipitated by adding equal volume of 7.5 M LiCl followed by incubation at −20°C for at least 1 hour. RNA pellets were washed with 70% ethanol and dissolved in nuclease-free water.

#### RT-PCR and qRT-PCR

Total RNAs were isolated from cells using TRIzol, as recommended, with an additional acidic phenol-chloroform (1:1) extraction step. The aqueous phase was precipitated with an equal volume of isopropanol, washed with 70% ethanol and rehydrated in 80 μl of nuclease-free water. RNA samples were then treated with 4-6 units of Turbo DNase (Ambion) at 37°C for 30 min to remove traces of genomic DNA, extracted with equal volume of acidic phenol-chloroform (1:1), precipitated with 3 volumes of 100% ethanol and 0.1 volume of 3 M sodium acetate (pH 5.2), washed with 70% ethanol and re-suspended with nuclease-free water. Reverse transcription (RT) was performed using SuperScript IV and random decamer (N10) primers at 50°C for 40 min. cDNA samples were analyzed by regular or quantitative PCR (qPCR). Regular PCR was done using ROCHE Taq DNA polymerase (Sigma-Aldrich, cat# 11596594001) and the RT-PCR products were resolved by electrophoresis in 1%–2% agarose gels. qPCR analyses were carried out using a Light Cycler®96 Real-Time PCR System (Roche) and qPCR BIO SyGreen Master Mix (PCR Biosystems; cat# PB20.16). The following primer combinations were used for RT-(q)PCR analyses of strRNAs:

##### PNCTR

RqP1 PNCTR_F1/PNCTR_R1
RqP2 PNCTR_F2/PNCTR_R2
RqP3 PNCTR_F3/PNCTR_R3
RP1 PNCTR_F4/PNCTR_R1
RP2 PNCTR_F4/PNCTR_R4
RP3 PNCTR_F2/PNCTR_R3

##### strRNA34

strRNA34_RqP1 strRNA34_F1/strRNA34_R1
strRNA34_RqP2 strRNA34_F2/strRNA34_R2
strRNA34_RqP3 strRNA34_F3/strRNA34_R3
strRNA34_RP1 strRNA34_F1/strRNA34_R3

##### strRNA79

strRNA79_RqP1 strRNA79_F1/strRNA79_R1
strRNA79_RqP2 strRNA79_F2/strRNA79_R2
strRNA79_RqP3 strRNA79_F3/strRNA79_R3
strRNA79_RP1 strRNA79_F1/strRNA79_R3

##### strRNA40

strRNA40_RqP1 strRNA40_F1/strRNA40_R1
strRNA40_RqP2 strRNA40_F2/strRNA40_R2
strRNA40_RqP3 strRNA40_F3/strRNA40_R3
strRNA40_RP1 strRNA40_F1/strRNA40_R3

##### strRNA91

strRNA91_RqP1 strRNA91_F1/strRNA91_R1
strRNA91_RqP2 strRNA91_F2/strRNA91_R2
strRNA91_RqP3 strRNA91_F3/strRNA91_R3
strRNA91_RP1 strRNA91_F1/strRNA91_R3

Other RT-(q)PCR primers are listed in Table S5. Unless mentioned otherwise, qRT-PCR signals were normalized to GAPDH mRNA expression levels (GAPDH_F1/GAPDH_R1; Table S5). In some experiments we used β-actin mRNA as an alternative normalization control (ACTB_F/ACTB_R; Table S5). To estimate PNCTR abundance, we analyzed total RNA prepared from 4,000 HeLa cells by qRT-PCR with PNCTR_F1/PNCTR_R1 primers and compared the signal with calibration curve obtained by qRT-PCR amplification of known amounts of a synthetic PNCTR RNA fragment transcribed from linearized pML154 (see above).

#### Northern blotting

RNA samples prepared as described in the previous section were separated by electrophoresis in 1.2% agarose gels containing 2.2 M formaldehyde, 40 mM MOPS (pH 7.0), 10 mM sodium acetate and 1 mM EDTA, partially hydrolyzed in 50 mM NaOH/1.5 M NaCl and transferred overnight to a Hybond N+ membrane in 10 × SSC as described (Brown et al., 2004). The membrane was UV-crosslinked (0.12 J/cm^2^), stained with methylene blue to visualize the 28S and 18S rRNAs, washed in 0.2 × SSC/1% SDS and blocked in ExpressHyb (Clontech; cat# 636831) at 68°C for 1 hour. Hybridization with heat-denatured DNA probe was done in ExpressHyb at 68°C for 2 hours. The membrane was washed twice in 2 × SSC, 0.05% SDS at 68°C and twice in 0.1 × SSC, 0.1% SDS at 50°C, 15 min each wash. The membrane was then dried and radioactive bands were visualized using a Typhoon Trio Variable Mode Imager (GE Healthcare).

#### Electrophoretic Mobility Shift Assays (EMSA)

EMSA was done as described previously (Rio, 2014, Zheng et al., 2012), with minor modifications. Briefly, 20 μL mixtures containing 20 mM HEPES-KOH (pH 7.9), 100 mM KCl, 2.2 mM MgCl_2_, 0.5 mM DTT, 0.2 mM EDTA, 20% (w/v) glycerol, and 50 nM [α-^32^P]-labeled PNCTR fragment were incubated for 30 min at 30°C with 0-1 μM purified recombinant PTBP1 or equal amounts of bovine serum albumin (BSA; Sigma-Aldrich; cat# 10711454001). For competition assays, 0-4 μM of unlabeled RNA probes were pre-incubated with 75 nM of PTBP1 protein for 30 min at 30°C before adding 50 nM of [α-^32^P]-PNCTR fragment probe and continuing the incubation for another 30 min. The RNA-protein complexes were separated by electrophoresis in 6% native polyacrylamide gels and the visualized using a Typhoon Trio Variable Mode Imager (GE Healthcare Life Sciences).

#### RNA immunoprecipitation (RIP)

Prior to cell lysis, 2.4 mg of Dynabeads Protein G (i.e., 80 μL of the original 30 mg/ml suspension; Thermo Fisher Scientific; cat# 10004D) were washed with 1 × PBS and 0.02% Tween-20, incubated with 6 μg of either PTBP1-specific antibody or non-immune mouse IgG in 500 μl of 1 × PBS/0.02% Tween-20 at room temperature for 1 hour with rotation and washed with 500 μl of 1 × PBS/0.02% Tween-20. To facilitate buffer exchange, beads were captured using a DynaMag-2 magnetic stand (Thermo Fisher Scientific, cat# 12321D). HeLa cells were grown in 15 cm dishes to 80%–90% confluency, washed once with ice-cold 1 × PBS, and scraped off in 2 ml/dish of ice-cold 10 mM Tris-HCl (pH 7.5), 150 mM NaCl, 0.5% NP40, 100 units/ml rRNasin (Promega; cat# N2111) and the recommended amount of cOmplete EDTA-free protease inhibitor cocktail (Sigma-Aldrich; cat# 04693132001). The lysates were incubated on ice for 30 min with occasional agitation and cleared by micro-centrifugation at 21,130 × g for 3 min at 4°C. 1 mL aliquots of the supernatant were mixed with 2.4 mg of drained antibody-loaded Dynabeads Protein G and rotated at 4°C for 150 min. Beads were subsequently washed with four changes of 1 × PBS/0.02% Tween-20, 250 μl each time. RNAs interacting with the beads were eluted with 1 mL of TRIzol, as recommended, and precipitated from the aqueous phase with 0.5 mL of isopropanol and 20 μg of purified glycogen (Sigma-Aldrich; cat# 10901393001) used as a carrier. RNA pellets were washed with 70% ethanol, rehydrated in nuclease-free water and analyzed by qRT-PCR using the following primers (see also Table S5).

RqP1 PNCTR_F1/PNCTR_R1
RqP2 PNCTR_F2/PNCTR_R2
U6 U6_F/U6_R
FOS FOS_F/FOS_R
PTBP2 PTBP2_RIP_F/PTBP2_RIP_R

PTBP1-, CELF1- and non-immune control antibody-derived RIP/qRT-PCR signals were normalized to no-antibody controls.

#### CLIP/qRT-PCR

UV-crosslinking and immunoprecipitation of RNA-protein complexes was carried out as described (Huppertz et al., 2014) with some modifications. HeLa cells were seeded into 10-cm dishes at ∼4 × 10^6^ cells/dish and allowed to attach overnight. The cells were then transfected with 400 nM gmControl or gmPNCTR for 24 hours, washed once with 10 mL of ice-cold 1 × PBS and irradiated with UV-C (Stratalinker 1800; 150 mJ/cm^2^; in 4 mL 1 × PBS on ice). PBS was aspirated and the cells were scraped off in 1 mL of CLIP lysis buffer [50 mM Tris-HCl, pH7.4, 100 mM NaCl, 1 mM MgCl_2_, 0.1 mM CaCl_2_, 1% Igepal CA-630 (Sigma Aldrich, cat# I8896), 0.1% SDS, 0.5% sodium deoxycholate and the recommended amount of cOmplete EDTA-free protease inhibitor cocktail]. The lysates was then sonicated using Bioruptor (Diagenode; low intensity settings, 5 cycles of 30 s on/ 30 s off; 4°C), incubated on ice for 5 min and treated with 4 μl Turbo DNase (Ambion; 2 units/μl) and 1 μl of murine RNase inhibitor (New Englands Biolabs; 40 units/μl) for 7 min at 37°C with shaking at 1100 rpm (Thermomixer compact, Eppendorf). To fragment RNA, 1 unit of RNase I (Thermo Fisher Scientific) was then added to the tubes and the shaking was continued for another 3 min at 37°C. The lysates were chilled on ice for 3 min and centrifuged at 21,130 × g for 15 min at 4°C to remove debris.

Beads for pre-clearing the lysates and immunoprecipitation were prepared as follow: 250 μl protein G dynabeads was pre-blocked with 0.1% BSA and 0.2 mg/ml yeast tRNA in 1 mL CLIP lysis buffer overnight at 4°C with rotation. Pre-blocked beads were washed once with CLIP lysis buffer and resuspended in 500 μl CLIP lysis buffer. Of the 500 μl, two 50-μl aliquots were set aside for pre-clearing the gmControl and the gmPNCTR lysates and four 100-μl aliquots were used for immunoprecipitations. Two 100-μl aliquots were incubated with 8 μg of the PTBP1-specific antibody and the other two, with 8 μg of the IgG control for 1 hour at room temperature with rotation. Antibody-conjugated beads were then washed once with 800 μl CLIP lysis buffer before proceeding to the next step.

The gmControl and the gmPNCTR lysates prepared as explained above were pre-cleared by incubating each of them with pre-blocked beads for 30 min at 4°C. To prepare input RNA samples, 10% aliquots of the pre-cleared lysates were set aside at this point and incubated with 1% SDS and 1 mg/ml of proteinase K (Thermo Fisher Scientific; Cat: EO0491) at 55°C for 30 min with shaking at 1100 rpm. The aliquots were then topped up with nuclease free water to 200 μl and extracted with 600 μl Trizol LS (Thermo Fisher Scientific; cat#10296010). After separating the aqueous and organic phases by the addition of 160 μl chroloform, we collected the top half of the aqueous phase, extracted it once with 300 μl of acidic phenol:chloroform (1:1) and once with 300 μl chloroform and precipitated with 8 μg glycogen, 0.3 M NaOAc, pH 5.2 and 1 volume of 100% isopropanol at −20°C overnight. The RNA pellets were finally washed with 70% ethanol and dissolved in nuclease-free water.

The remaining 90% of the pre-cleared gmControl and the gmPNCTR lysates were split into two equal aliquots, which were incubated with PTBP1- and IgG-conjugated beads, respectively, overnight at 4°C with rotation. Next morning, the beads were washed three times with ice-cold 50 mM Tris-HCl, pH 7.4, 1 M NaCl, 1 mM EDTA, 1% Igepal CA-630, 0.1% SDS, 0.5% sodium deoxycholate, twice with ice-cold 20 mM Tris-HCl, pH 7.4, 10 mM MgCl_2_, 0.2% Tween-20 and once with proteinase K buffer (100 mM Tris-HCl, pH 7.4, 50 mM NaCl, 10 mM EDTA). Immunoprecipitated RNA fragments were then eluted by incubating the beads with 200 μl of proteinase K buffer additionally supplemented with 1% SDS and 1 mg/ml of proteinase K (Thermo Fisher Scientific; Cat: EO0491) at 55°C for 30 min. The mixtures were extracted once with 200 μl of acidic Phenol: chloroform (1:1) and once with 200 μl chloroform and precipitated with 20 μg glycogen, 0.1 M NaCl, 0.3 M NaOAc, pH 5.2 and 3 volumes of 100% ethanol at −80°C for 1 hour. The RNA pellets were washed once with 70% ethanol, resuspended in nuclease free-water and analyzed alongside the input samples by qRT-PCR using the following primers (see also Table S5):

CLIP-CHEKe2 CLIP-CHEKe2_F/CLIP-CHEKe2_R
CLIP-CHEKe8 CLIP-CHEKe8_F/CLIP-CHEKe8_R
CLIP-CHEKi8 CLIP-CHEKi8_F/CLIP-CHEKi8_R
CLIP-PTBP2e10 CLIP-PTBP2e10_F/CLIP-PTBP2e10_R
ACTB ACTB_F/ACTB_R

#### CAP trapper assays

To test if the 5′ end of PNCTR was modified by a guanosine triphosphate-based cap, we used a modified version of the CAP trapper protocol (Carninci et al., 1996). We first oxidized vicinal 2′,3′-diol groups present in most caps and 3′-terminal RNA nucleotides by incubating 12 μg of total RNA in 50 μl of 66 mM NaOAc (pH 5.2) and freshly prepared 5 mM sodium periodate for 1h on ice in the dark. A negative control reaction was set up in a similar manner but without sodium periodate. The oxidized and control RNAs were precipitated with 3.3 μL of 7.5 M LiCl, 1 μL of 10% SDS and 50 μl of isopropanol for 30 min at −20°C and centrifuged at 21,130 × g for 15 min at 4°C. The RNA pellets were washed once with 70% ethanol and dissolved in 50 μl of nuclease-free water. The solutions were then supplemented with 5 μL of 1M NaOAc (pH 6.1), 5 μL of 10% SDS and 150 μL of freshly dissolved 10 mM biocytin hydrazide and incubated overnight at room temperature in the dark to biotinylate periodate-oxidized groups. The RNAs were precipitated from biotynylation mixtures with 5 μL of 5 M NaCl, 75 μL of 1 M NaOAc (pH 6.1) and 725 μL of 100% ethanol, incubated on ice for 1 hour, spun at 21,130 × g for 15 min at 4°C, washed once with 70% ethanol, once with 80% ethanol and dissolved in 22 μl of nuclease-free water.

The RNAs were used as templates for first-strand cDNA synthesis using SuperScript IV and N10 primers as described above. The RT reactions (40 μl) were then supplemented with 20 μL of 100 mM Tris-HCl (pH 7.5), 1 M NaCl and 50 mM EDTA and 140 μL of nuclease-free water and treated with 250 units of RNase I for 30 min at 37°C to digest RNA sequences that are not base-paired with cDNA. The RNA-cDNA duplexes were precipitated by 5 μL of 10% SDS, 5 μL of 5 M NaCl, 75 μL of 1 M NaOAc (pH 6.1), 0.5 μL of glycogen (20 μg/μl) and 720 μL of 100% ethanol and incubated for 1 hour at −20°C. The RNA-cDNA duplexes were washed once with 70% ethanol, dissolved in 55 μl of ice-cold Elution buffer (10 mM Tris-HCl, pH 7.5 and 0.1 mM EDTA) and mixed with 55 μl of 2 × Wash/Binding buffer (1 M NaCl, 40 mM Tris-HCl, pH 7.5, 2 mM EDTA) before proceeding to the next step.

At this point, cDNA-RNA duplexes corresponding to the 5′ ends of capped RNAs should be covalently modified by biotin groups in the periodate-oxidized/biocytin hydrazide-treated sample (but not in the negative control). To capture these biotinylated duplexes, we used hydrophilic streptavidin magnetic beads (New England Biolabs, cat#S1421S) prepared in the following manner. For each RNA-cDNA sample, 1 mg (250 μl) of Streptavidin beads was washed twice with 500 μl of Wash/Binding buffer (0.5 M NaCl, 20 mM Tris-HCl, pH 7.5, 1 mM EDTA). The beads were blocked with 100 μl of Wash/Binding buffer containing 10 μg/μl of yeast tRNA at room temperature for 1 hour and washed twice with 500 μl of Wash/Binding buffer. The beads were then combined with the RNA-cDNAs mixtures prepared as described above and incubated for 10 min at room temperature with occasional agitation. The beads were washed twice with 500 μl Wash/Binding buffer at room temperature, once with ice-cold Low-Salt Wash buffer (0.15 M NaCl, 20 mM Tris-HCl, pH 7.5 and 1 mM EDTA), and twice with ice-cold Elution buffer. The cDNAs were then eluted in 125 μl of Elution buffer at 96°C for 5 min followed by quickly separating the eluate from the beads using a DynaMag-2 magnetic stand. The elution step was repeated once and the two eluates containing cDNA copies of capped RNAs were pooled, extracted with a 1:1 mixture of Tris-HCl-equilibrated phenol:chloroform and precipitated with 3 volumes of 100% ethanol, 0.1 volume of 3 M sodium acetate (pH 5.2) and 0.6 μl of 20 μg/μl glycogen. The cDNAs were rehydrated in nuclease-free water and analyzed by qPCR with the following primer pairs appropriate for detection of 5′-proximal RNA sequences (see also Table S5):

PNCTR PNCTR_F0/PNCTR_R0
47S/45S 45S_F/45S_R
ACTB ACTB_F/ACTB_R
GAPDH GAPDH_F2/GAPDH_R2
U6 U6_F/U6_R

#### Analysis of RNA polyadenylation status

Polyadenylated RNA fraction was isolated using the MagJET mRNA Enrichment Kit (Thermo Fisher Scientific, cat# K2811), as recommended. Briefly, 50 μg of total HeLa RNA was incubated at 65°C for 5 min and chilled on ice for 2 min. The RNA was then mixed with pre-washed MagJET oligo(dT) beads (50 μl of beads washed twice with 50 μl hybridization buffer and resuspended in 100 μl of hybridization buffer) and incubated at room temperature with gentle agitation for 5 min followed by separation of the beads from the supernatant using a DynaMag-2 magnetic stand. The supernatant containing the non-polyadenylated RNA fraction (“flow-through”) was collected and the beads were washed 3 times with the wash buffer. The beads were then resuspended in 50 μl of nuclease-free water, heated at 60°C for 2 min and then cooled to room temperature for 5 min. This was followed by the addition of 50 μl of hybridization buffer and a 5-min incubation at room temperature to recapture polyadenylated RNAs. The beads were then washed twice with the wash buffer, resuspended in 50 μl nuclease-free water, incubated at 60°C for 2 min and then immediately separated from the eluate containing polyadenylated RNAs using DynaMag-2. Both the flow-through and the oligo(dT) bead-bound fractions were then analyzed by qRT-PCR with the following primers (see also Table S5).

RqP1 PNCTR_F1/PNCTR_R1
RqP2 PNCTR_F2/PNCTR_R2
RqP3 PNCTR_F3/PNCTR_R3
47S/45S 45S_F/45S_R
ACTB ACTB_F/ACTB_R
GAPDH GAPDH_F1/GAPDH_R1
U6 U6_F/U6_R

#### RNA-seq

HeLa cells were plated overnight in a 12-well plate at 2 × 10^5^/well in 1 mL of DMEM with 10% FBS without antibiotics. Next morning, cells were transfected with 25 pmol/well of an appropriate gapmer (QIAGEN) that was mixed with 3 μl of Lipofectamine RNAiMAX pre-diluted in 100 μl of OPTI-MEM. Total RNAs were extracted 24 hours post transfection using TRIzol and a PureLink RNA Mini Kit with on-column PureLink DNase (Thermo Fisher Scientific; cat# 12185010) treatment, according to the manufacturer’s recommendations. The RNAs were eluted in nuclease-free water, QC’d (Bioanalyzer RIN = 9.8) and hybridized with oligo(dT) magnetic beads to isolate the poly(A) RNA fraction used for subsequent library preparation steps. Stranded mRNA sequencing libraries were prepared using the TruSeq Stranded mRNA Library Preparation Kit (Illumina cat## RS-122-2101 and RS-122-2102). Purified libraries were qualified on an Agilent Technologies 2200 TapeStation using a D1000 ScreenTape assay (cat## 5067-5582 and 5067-5583). The molarity of adaptor-modified molecules was defined by quantitative PCR using the Kapa Library Quant Kit (Kapa Biosystems; cat# KK4824). Individual libraries were normalized to 10 nM and equal volumes were pooled in preparation for Illumina sequence analysis. Sequencing libraries (25 pM) were chemically denatured and applied to an Illumina HiSeq v4 single read flow cell using an Illumina cBot. Hybridized molecules were clonally amplified and annealed to sequencing primers with reagents from a HiSeq SR Cluster Kit v4-cBot (Illumina; cat# GD-401-4001). Following transfer of the flowcell to a HiSeq 2500 instrument (Illumina; cat## HCSv2.2.38 and RTA v1.18.61), a 50 cycle single-read sequence run was performed using HiSeq SBS Kit v4 sequencing reagents (Illumina; cat# FC-401-4002). All library preparation and sequencing steps were carried out by the Huntsman Cancer Institute High-Throughput Genomics facility, University of Utah, USA.

#### Immunoblotting

Cells were washed three times with ice-cold 1 × PBS and proteins were extracted using RIPA lysis buffer (Santa Cruz Biotechnology; cat# sc-364162) supplemented with 1 mM PMSF and the recommended amount of cOmplete EDTA-free protease inhibitor cocktail. Protein concentrations were determined using a Pierce BCA Protein Assay Kit. Protein samples (10-20 μg) were then incubated at 95°C for 5 min in 1 × Laemmli sample buffer (50 mM Tris-HCl, pH 6.8, 100 mM DTT, 2% SDS, 10% glycerol and 0.1% bromophenol blue), separated by 4%–20% gradient SDS-PAGE (Bio-Rad; cat# 4561096), electrotransferred to nitrocellulose membranes and analyzed using appropriate primary and secondary antibodies. Fluorescent immunoblot signals were detected using Odyssey imaging system (LI-COR Biosciences). Enhanced chemiluminescence (ECL) detection was done using reagents from Thermo Fisher Scientific (ECL kits cat## 32106 or 11546345 and GE Healthcare Amersham Hyperfilm cat# 10607665). Protein band intensities were quantified using LI-COR Image Studio software (LI-COR Biosciences). To estimate PTBP1 abundance, lysates from 1 × 10^5^ HeLa cells were analyzed by immunoblotting alongside known amounts of His-tagged PTBP1 purified from bacteria.

#### Immunofluorescence combined with RNA fluorescence *in situ* hybridization (IF-FISH)

HeLa cell cultures were grown on 18 mm round coverslips pre-coated with 50 μg/ml poly-D-lysine. The coverslips were washed with 1 × PBS, incubated with 100 mM NaCl, 300 mM sucrose, 10 mM PIPES (pH 7.8), 3 mM MgCl_2_, 0.5% Triton X-100, 80 units/ml murine RNase inhibitor (New England Biolabs; cat# M0314) for 4 min on ice and fixed with 4% paraformaldehyde (Ted Pella; cat# 18501) for 15 min at room temperature. The coverslips were then washed three times with 1 × PBS, blocked with IF-FISH blocking buffer [1 × PBS containing 0.5% BSA (Thermo Fisher Scientific; cat# BP8805) and 0.2% Tween-20 (Sigma-Aldrich; cat# P9416)] for 30 min at room temperature and then incubated with appropriate primary antibodies in the IF-FISH blocking buffer additionally containing 20 units/ml murine RNase inhibitor (New England Biolabs; cat# M0314) for 16-18 hours at 4°C. Following three washes with 1 × PBS the coverslips were then incubated with corresponding Alexa Fluor-conjugated secondary antibodies for 1 hour at room temperature. The coverslips were then washed three times with 1 × PBS and post-fixed with 4% paraformaldehyde for 15 min at room temperature, washed three times with 1 × PBS and used for subsequent RNA-FISH staining.

To perform RNA-FISH, 70 ng of biotinylated PNCTR FISH probe prepared as described above was co-precipitated with 5 μg human Cot1-DNA (Thermo Fisher Scientific; cat# 15279011), 5 μg salmon sperm DNA and 10 μg yeast tRNA (Sigma-Aldrich; cat# R8508) using 3 volumes of ethanol and 0.1 volume of 3 M sodium acetate (pH 5.2). The probe pellet was washed with 70% ethanol, dissolved in 15 μl of 100% formamide, denatured at 65°C for 10 min and chilled on ice. The probe was then mixed with 15 μl of 2 × FISH hybridization buffer [0.4% BSA, 4 × SSC, 20% dextran sulfate (Sigma-Aldrich, cat# D8906), 40 mM Ribonucleoside Vanadyl Complex (Promega, cat# S1402S)] and incubated with cells overnight at 37°C. The coverslips were then washed once with 50% formamide and 2 × SSC at 37°C, once with 2 × SSC at 37°C and once with 1 × SSC at room temperature, 15 min each wash, and once in 4 × SSC for 1-2 min. This was followed by incubating the coverslips with Alexa Fluor 647-conjugated streptavidin in 4 × SSC, 0.8% BSA and 0.8 units/μl murine RNase inhibitor (New England Biolabs; cat# M0314) for 1 hour at 37°C, and washing them at room temperature once with 4 × SSC, once with 4 × SSC and 0.1% Triton X-100 and once with 4 × SSC, 10 min each wash. The coverslips were finally stained with 0.5 μg/ml DAPI and mounted onto microscope slides using ProLong Gold antifade reagent (Thermo Fisher Scientific, cat# P36934). A similar protocol was used for formalin-fixed paraffin-embedded tissue sections except the IF part was replaced by dewaxing, rehydration, and permeabilization steps (https://biosearchassets.blob.core.windows.net/assets/bti_stellaris_protocol_ffpe_tissue.pdf). Images were taken using an LSM 800 confocal microscope (Zeiss) equipped with a 63 × Plan- Apochromat, 1.4 NA oil immersion objective. To quantify PNCTR and PTBP1 dots, Z stacks were taken at 0.35 μm intervals. Maximum intensity projections of the Z series were thresholded, converted to a binary format and used as an input for the “Analyze Particles” application of ImageJ.

#### Immunofluorescence combined with single-molecule RNA fluorescence *in situ* hybridization (IF-smFISH)

HeLa cell cultures were grown on 18 mm round coverslips and fixed with 4% paraformaldehyde as described in the IF-FISH section above. The coverslips were then washed three times with 1 × PBS, blocked with IF-smFISH blocking buffer [1 × PBS containing 0.5% BSA (Thermo Fisher Scientific; cat# BP8805)] for 30 min at room temperature and incubated with appropriate primary antibodies in the IF-smFISH blocking buffer additionally containing 20 units/ml murine RNase inhibitor (New England Biolabs; cat#M0314) for 1 hour at room temperature. Following three washes with 1 × PBS, the coverslips were incubated with corresponding Alexa Fluor-conjugated secondary antibodies for 1 hour at room temperature. The coverslips were then washed three times with 1 × PBS and post-fixed with 4% paraformaldehyde for 10 min at room temperature, washed three times with 1 × PBS and used for subsequent RNA-smFISH staining.

The coverslips were washed once with Wash Buffer A [20% Stellaris RNA FISH Wash Buffer A (Biosearch Technologies; cat# SMF-WA1-60) and 10% formamide in nuclease-free water] for 5 min at room temperature, and incubated with Stellaris RNA FISH Hybridization Buffer (Biosearch Technologies; cat# SMF-HB1-10) containing 10% formamide and 125 nM of the PNCTR-specific Stellaris probe in the dark at 37°C overnight. The coverslips were then washed once with Wash Buffer A and once with Wash Buffer A containing 0.1 μg/ml DAPI, both washes in the dark at 37°C for 30 min, and mounted onto microscope slides using ProLong Gold antifade reagent.

Images were taken and maximum intensity projections produced as described in the IF-FISH section. Diffraction-limited single-molecule signals were selected manually and their background-subtracted intensities were quantified using ImageJ. The total number of PNCTR molecules per nucleus was then estimated by dividing the overall PNCTR signal intensity by the median intensity of single-molecule signals. PTBP1 sequestration was estimated as a fraction of total nuclear PTBP1 minus cytoplasmic background co-localizing with the PNCTR signal.

#### Clonogenic assays

HeLa cells were transfected with 25-400 nM of gmControl or gmPNCTR as described above. 24 hours post transfection cells were trypsinized, re-plated into 6-well plates at 100-10,000 cells/well and incubated for 10 days changing the medium every 2-3 days. Cell colonies were stained with 0.1% methylene blue in 50% (v/v) methanol for 1 hour at room temperature, washed with water and air-dried. Confluency of each well was then quantified using ImageJ.

#### Cell viability assays

HeLa cells were plated into 96-well plates at 1 × 10^4^/well. Next morning, the cells were treated with 25-400 nM gmControl or gmPNCTR for up to 72 hours. Cell viability in the transfected wells was assayed using PrestoBlue Cell Viability Reagent according to the manufacturer’s recommendation. Briefly, 0.1 volume of PrestoBlue reagent was added directly to culture medium and incubated at 37°C for 30 min. After incubation, absorbance reads were taken at both 570 nm and 630 nm (as a reference wavelength) using an MRX II microplate reader (Dynex Technologies). The experimental values were acquired by normalization of 570 nm to 630 nm to plot the graph. Two independent experiments were carried out with each condition tested in triplicate (i.e., 6 repeats in total).

#### Bioinformatics

To predict transcripts with multiple RBP motifs, ENCODE RNA-seq reads for 5 commonly used human cell lines, A549, HeLa (clone S3), HepG2, K562 and MCF7 (see the list of Deposited Data above) were aligned with TopHat2 using a GRCh38.p3-based Bowtie2 genome index and a GENCODE v23 GTF human transcriptome annotation file, as follows:

tophat -p <n_threads> -o <out_dir>–library-type fr-firststrand \

-G <GENCODEv23_transcriptome_gtf_file> \

–transcriptome-index <bowtie2_transcriptome_index> \

<bowtie2_genome_index> PE_reads_1.fastq PE_reads_2.fastq

BAM files generated by TopHat2 were used for subsequent GENCODE v23 reference-guided transcriptome assembly done using StringTie with relaxed muli-mapping read settings:

stringtie -p <n_threads> -M 1 -o <out_gtf_file> \

-G <GENCODEv23_transcriptome_gtf_file> \

<TopHat2_accepted_hits_bam_file>

GTF files produced by StringTie were merged into a single transcriptome file:

cuffmerge -p <n_threads> -o <out_dir> \

–min-isoform-fraction 0.2 \

-g <GENCODEv23_transcriptome_gtf_file> \

<GTF_list.txt>

We applied two filters to the merged transcriptome file to reduce the incidence of false positives. First, we removed all transcripts that did not overlap known transcribed sequences (a combination of exonic ranges in GENCODE v23 GTF and mRNA and EST sequences downloaded from https://genome.ucsc.edu/cgi-bin/hgTables) at least by one nucleotide in a strand-specific manner:

bedtools intersect -s -sorted -split -u \

-a <sorted_bed12_for_merged_transcriptome> \

-b <sorted_bed6_for_GENCODEv23_mRNA_EST_exons>

Second, we discarded transcripts expressed at relatively low levels across the 5 cancer lines (median < 0.1 TPM) according to Kallisto, a program allocating RNA-seq reads (including multi-mapping ones) to their likely RNA origins using an expectation-maximization algorithm:

kallisto quant -i <Kallisto_transcriptome_index> \

-o <out_dir> -b 1 PE_reads_1.fastq PE_reads_2.fastq

Novel transcripts passing the two above filters and all the GENCODE v23 transcripts were combined into a single transcriptome annotation and used to calculate the occurrence of known RBP motifs downloaded as position weight matrices (PWMs) from CisBP-RNA (http://cisbp-rna.ccbr.utoronto.ca/; *Homo sapiens*, November 23, 2015 freeze). PWM matches were identified using the FIMO package of the MEME suite and a final transcriptome-based Markov background file:

fasta-get-markov <final_transcriptome_fasta_file> markov1.b -norc -m 1

fimo–no-qvalue–norc–thresh 0.001–motif-pseudo 0.1 \

–max-stored-scores 100000000–bgfile markov1.b–oc <out_dir> \

<MEME_formatted_PWM_file> <final_transcriptome_fasta_file>

In addition to imposing the FIMO threshold of p < 0.001, we discarded matches with < 0.85 fit to the maximally achievable PWM score as described (Pan and Phan, 2008). For each PWM, we calculated z-scores for numbers and densities of qualifying matches in R. Candidate transcripts with both z-scores ≥ 5 (n = 251) were retained for further analyses. Of these, 96 were classified as “unknown intergenic RNAs” (StringTie class code “u”; Table S1). hg38-specific STRs were downloaded from https://genome.ucsc.edu/cgi-bin/hgTables (Simple Tandem Repeats by TRF), merged into a bed file containing non-overlapping intervals and intersected with strRNA and control sequences using Bedtools. The longest ORF was predicted for each strRNA by TransDecoder using the entire cancer cell-specific transcriptome with median expression ≥ 0.1 TPM and all known protein-coding transcripts from GENCODE v23 as controls.

Possible overlap of strRNAs with lncRNA entries from the LNCipedia database (https://lncipedia.org/downloads/lncipedia_5_0_hc_hg38.bed) was analyzed using Bedtools:

bedtools intersect -s -split -wo -f 0.25 \

-a <strRNA_bed_file> -b <lncipedia_bed_file>

To compare splicing events regulated by PNCTR and PTBP1/PTBP2, we analyzed corresponding datasets generated in this study (ArrayExpress; E-MTAB-6529) and published previously (Ling et al., 2016) using two alternative approaches. In the first approach, we identified regulated skipped exons by processing RNA-seq data using ExpressionPlot and hg18-derived annotation files provided with this package. The coordinates of regulated exons were mapped to the GRCh38/hg38 assembly using the USCS Genome Browser liftOver program (https://genome.ucsc.edu/cgi-bin/hgLiftOver). Alternatively, RNA-seq reads were aligned to the hg19 assembly using HISAT2 with a premade index (ftp://ftp.ccb.jhu.edu/pub/infphilo/hisat2/data/hg19.tar.gz) and coordinates of known splice sites extracted from an hg19 transcriptome annotation file (http://genes.mit.edu/burgelab/miso/annotations/ucsc_tables/hg19/ensGene.gff3). After converting HISAT2-generated SAM files into sorted and indexed BAM files with Samtools we identified regulated exonic events using MISO and annotations for skipped exons, mutually exclusive exons and alternative 5′ and 3′ splice sites downloaded from http://genes.mit.edu/burgelab/miso/annotations/ver2/miso_annotations_hg19_v2.zip. In both cases, statistical significance of overlaps between different sets of regulated exons was calculated by analyzing a contingency table with regulated and non-regulated or up- and downregulated categories using Fisher’s exact test.

To analyze genome-wide binding patterns of pol I, publicly available ChIP-seq data [(Sanij et al., 2015); GEO: GSM1544525] for the hTERT-immortalized human mammary epithelial cell line (HMEC) were aligned to the GRCh38.p3 genome using Bowtie2 with default parameters. After removing duplicated reads and reads with alignment quality AS < −2, pol I genomic distribution was visualized using Integrative Genomics Viewer (IGV). BED files with PTBP1-specific CLIP-seq and iCLIP clusters for HeLa cells were downloaded from previously published studies [(Xue et al., 2009); GEO: GSE19323; (Coelho et al., 2015, Haberman et al., 2017); ArrayExpress: E-MTAB-3108; https://github.com/jernejule/non-coinciding_cDNA_starts/blob/master/HeatMaps_of_PTBP1-motifs_around-eCLIP-iCLIP-irCLIP-clusters-PTBP1/data/PTBP1-iCLIP1.3nt.peaks.3nt.clusters.bed.gz] and mapped to GRCh38/hg38 using the USCS Genome Browser liftOver tool. To identify high-confidence PTBP1-RNA interaction sites, we discarded clusters not intersectable within the monomeric and dimeric duplicates of the CLIP-seq dataset and merged the remaining monomeric and dimeric clusters into a single track. The iCLIP clusters were used without further modifications.

### Quantification and Statistical Analyses

All statistical procedures were carried out using Microsoft Excel and R and, unless stated otherwise, experimental data were averaged from at least three experiments and shown with error bars representing SD. Data obtained from qRT-PCR, immunoblot quantifications, colony formation and cell viability assays were typically analyzed using a two-tailed Student’s t test assuming unequal variances. RT-PCR and immunoblot quantification data for series of independently generated pairs of control and experimental treatments were analyzed using paired t test. Immunofluorescence data were analyzed using a two-sided Kolmogorov-Smirnov (KS) test. Numbers of experimental replicates and p values are provided in the figures.

### Data and Software Availability

RNA-seq data generated in this study are available from ArrayExpress: E-MTAB-6529.

## Supporting Citations

The following reference appears in the Supplemental Information: Moraes et al., (2006).

## Supplemental Information

Document S1. Figures S1–S7

Table S1. strRNAs Predicted in This Study, Related to Figure 1

Table S2. PNCTR and PTBP1/PTBP2-Regulated Exons, Related to Figure 6

Table S3. Patient Samples Used in This Work, Related to STAR Methods

Table S4. Plasmids Generated in This Study, Related to STAR Methods

Table S5. DNA Oligonucleotides Used in This Study, Related to STAR Methods

Document S2. Article plus Supplemental Information
