# Supplementary material for: A Short Tandem Repeat-Enriched RNA Assembles a Nuclear Compartment to Control Alternative Splicing and Promote Cell Survival
Source: Mol Cell. 2018 Nov 1;72(3):525–540.e13. doi: 10.1016/j.molcel.2018.08.041 (PMC6224606; doi:10.1016/j.molcel.2018.08.041)
Supplement: Document S1. Figures S1–S7 [file mmc1.pdf]

Molecular Cell, Volume *72*

Supplemental Information

A Short Tandem Repeat-Enriched RNA

Assembles a Nuclear Compartment to Control

Alternative Splicing and Promote Cell Survival

Karen Yap, Svetlana Mukhina, Gen Zhang, Jason S.C. Tan, Hong Sheng Ong, and Eugene V. Makeyev

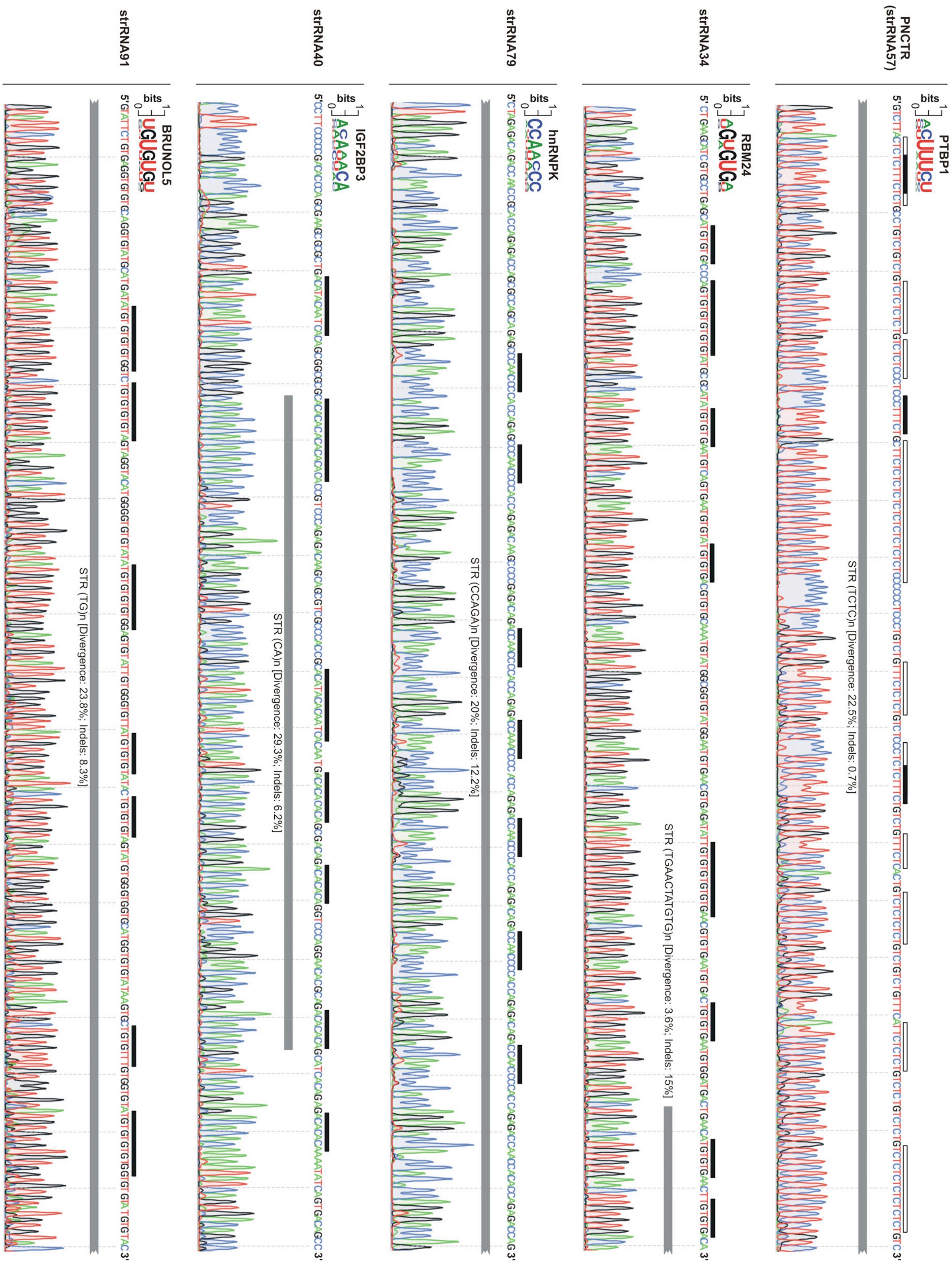

Figure S1. Sanger sequencing results for five strRNAs selected for validation, Related to Figure 1.

Filled boxes on the top of each sequence indicate motifs with strong similarity to RBP-specific PWMs from the CISBP-RNA database [http://cisbp-rna.ccbr.utoronto.ca/; (Ray et al., 2013)]. The PWMs are also shown as RNA sequence logos. Open boxes on the top of the PNCTR/strRNA57 sequence correspond to the YUCUYY and the YYUCUY motifs based on PTBP1 in vivo binding preferences (Llorian et al., 2010). Gray boxes below the sequences show corresponding STR elements from the UCSC Genome Browser database.

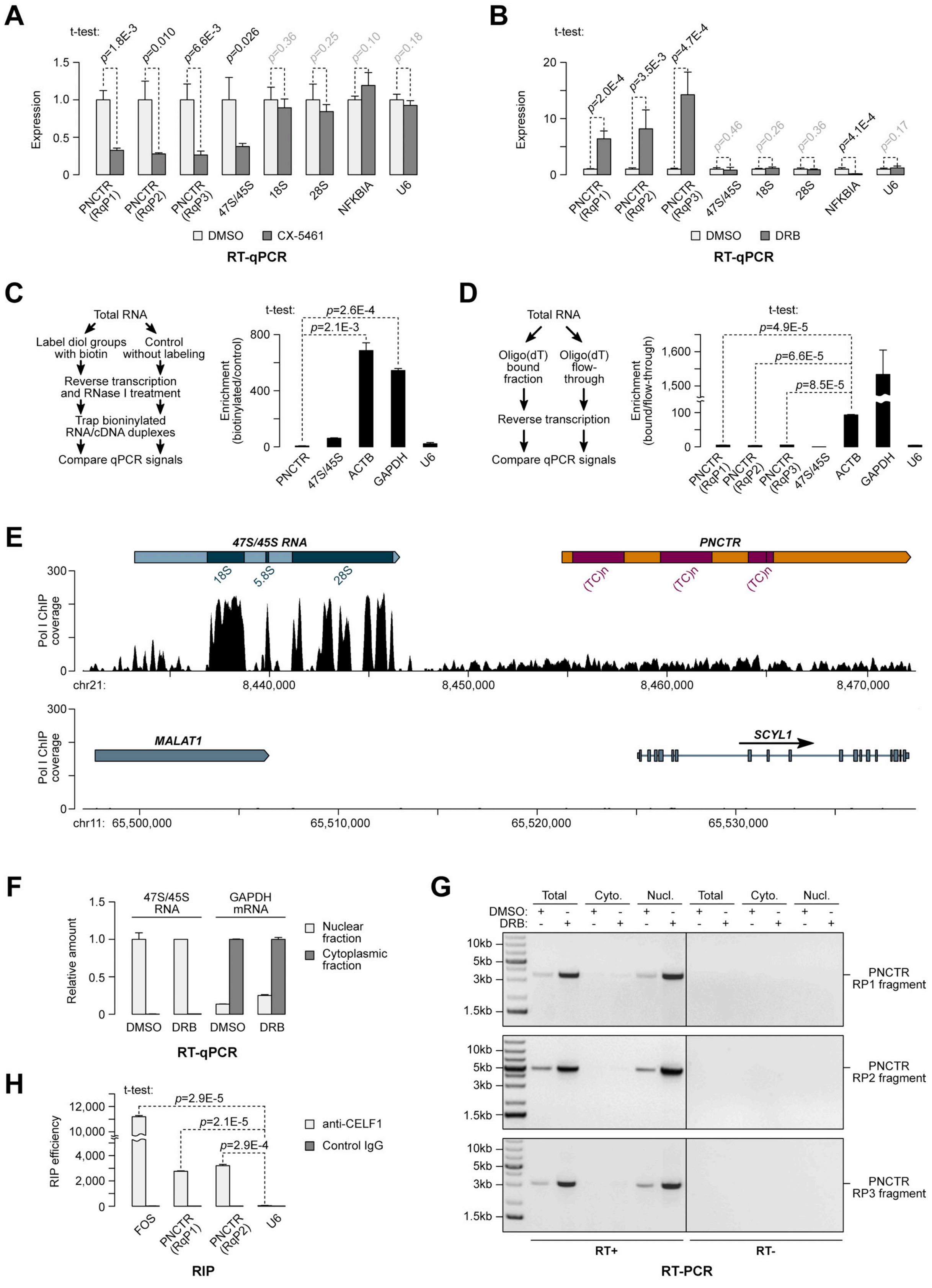

Figure S2. Initial characterization of the strRNA PNCTR, Related to Figure 2.

(**A**) HeLa cells were incubated for 6 hours with the pol-I inhibitor CX-5461 or DMSO and analyzed by RT-qPCR. Note that CX-5461 down-regulates PNCTR (based on the data obtained using three different primer pairs, RqP1, RqP2 and RqP3). As expected, it also down-regulates the 47S/45S rRNA precursor. However, the levels of mature 18S and 28S rRNAs do not change significantly during this relatively short incubation, probably because these RNAs have longer half-lives than PNCTR and 47S/45S. The expression of pol-II (NFKBIA mRNA) and pol-III transcripts (U6 snRNA) is also not affected.

(**B**) RT-qPCR analysis of HeLa cells incubated for 6 hours with the pol-II inhibitor DRB or DMSO showing that DRB dampens the expression of the NFKBIA mRNA but has no effect on 47S/45S, 18S, 28S and U6 transcripts. Surprisingly, it increases PNCTR expression (RqP1, RqP2 and RqP3 primer pairs) through a yet-to-be-understood mechanism.

(**C-D**) CAP trapper (C) and oligo(dT) pull-down (D) assays suggesting that PNCTR lacks a guanosine triphosphate cap and a poly(A) tail, as expected for a pol-I transcript. ACTB and GAPDH mRNAs are used as positive and 47S/45S rRNA and U6 snRNAs as negative controls. See STAR Methods for further details.

(**E**) *Top*, consistent with its role in PNCTR transcription, pol-I crosslinks are readily detectable in the corresponding IGS region in a publicly available ChIP-seq dataset for an immortalized HMEC cell line (Sanij et al., 2015). *Bottom*, virtually no pol-I crosslinks are found in a control genomic region encoding the pol II-transcribed lncRNA MALAT1 [Metastasis Associated Lung Adenocarcinoma Transcript 1; (Sun et al., 2017)].

(**F**) Quality of nuclear and cytoplasmic fractions prepared from DMSO and DRB-treated HeLa cells was confirmed by RT-qPCR analysis of RNAs known to be

enriched in the nucleus (47S/45S) and the cytoplasm (GAPDH mRNA). Data are averaged from 3 amplifications ±SD and the expression levels of 47S/45S in the nucleus and GAPDH in the cytoplasm are set to 1.

(**G**) PNCTR expression in whole cells (Total) and their cytoplasmic (Cyto.) and nuclear (Nucl.) fractions was analyzed by semi-quantitative RT-PCR. Note that PNCTR localizes almost exclusively to the nucleus and that the up-regulation of this strRNA by DRB (see panel B) does not alter its nucleocytoplasmic distribution. Also note that the PCR products are detected only in the presence of RT (RT+) but not in its absence (RT-) suggesting that the amount of genomic DNA contaminants in our samples is negligible.

(**H**) RIP analysis showing that PNCTR forms detectable physical contacts with CELF1, a well-known PNC marker (Norton and Huang, 2013). RNAs immunoprecipitated with a CELF1-specific antibody or a non-immune IgG control were analyzed by RT-qPCR using primers for PNCTR, FOS mRNA (positive control; (Moraes et al., 2006)) or U6 snRNA (negative control).

Data in (A-D, F, and H) are averaged from three experiments ±SD and compared (A-D, H) by a two-tailed t-test assuming unequal variances.

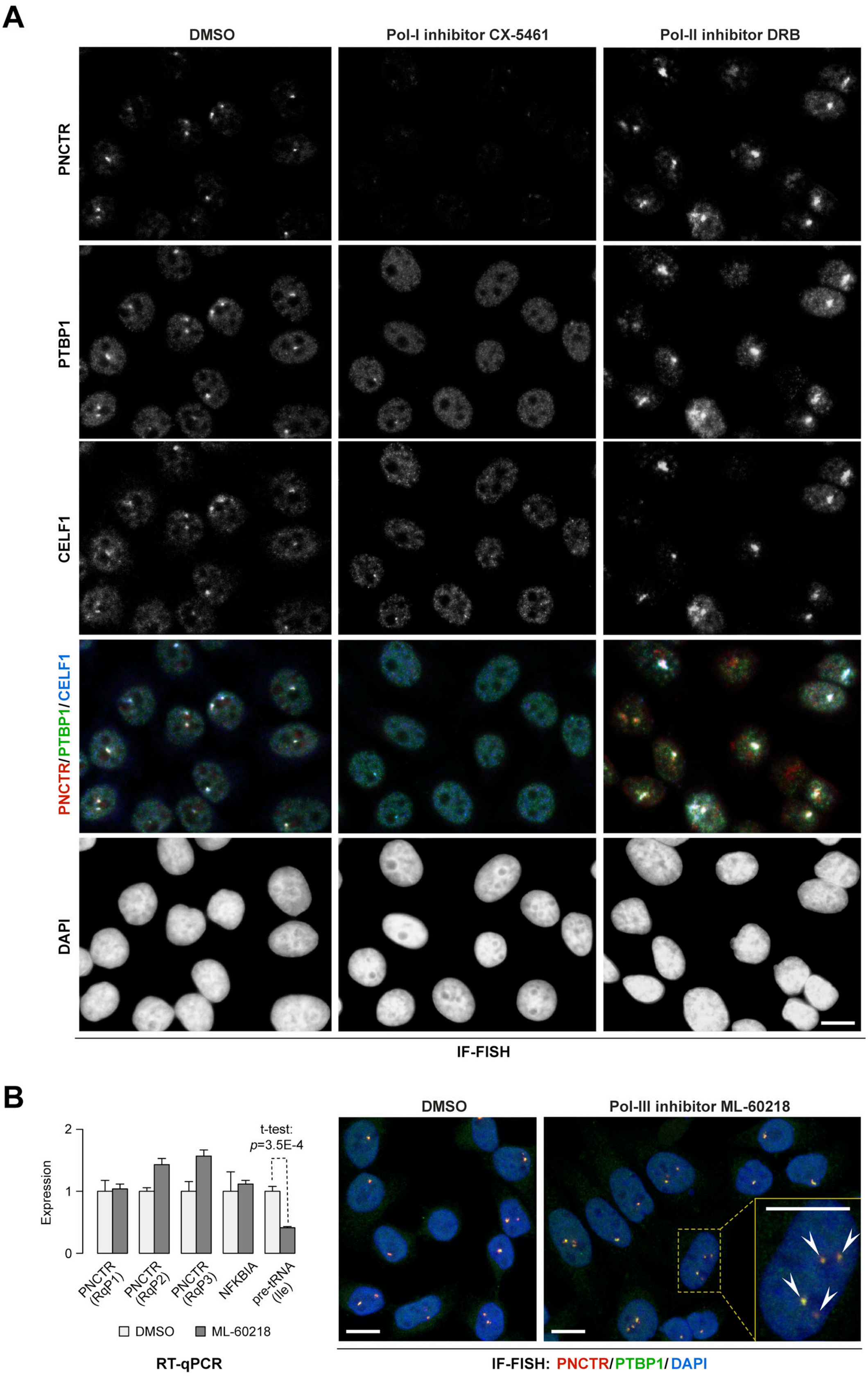

Figure S3. Effect of RNA polymerase inhibitors on the PNC, Related to Figure 3.

(**A**) HeLa cells treated with DMSO, CX-5461 or DRB for 6 hours were co-stained with a PNCTR-specific FISH probe and antibodies against the PNC markers PTBP1 and CELF1. In line with the inhibitor data in Fig. 2B and Fig. S2, PNCTR foci virtually disappear after treating HeLa cells with CX-5461 and increase in size in response to DRB. These effects are mirrored by the corresponding changes in the PTBP1- and CELF1-positive foci suggesting that PNCTR might be required for PNC assembly.

(**B**) HeLa cells were treated with either DMSO or the pol-III inhibitor ML-60218 for 6 hours and analyzed by RT-qPCR (*left*) and IF-FISH (*right*). Confirming ML-60218 specificity, it does not change expression of PNCTR or NFKBIA mRNA but dampens the levels of an intron-containing tRNA precursor, pre-tRNA(Ile), transcribed by pol III. Note that many ML-60218-treated cells have phenotypically normal PNC dots. In a subset of cells, however, ML-60218 appears to cause partial PNC fragmentation with 3-4 smaller dots (marked by arrowheads in the close-up image) detectable instead of the usual 1-2. This is generally consistent with the previously reported function of pol-III transcription in PNC integrity (Norton and Huang, 2013). Scale bars in (A-B), 10 μm.

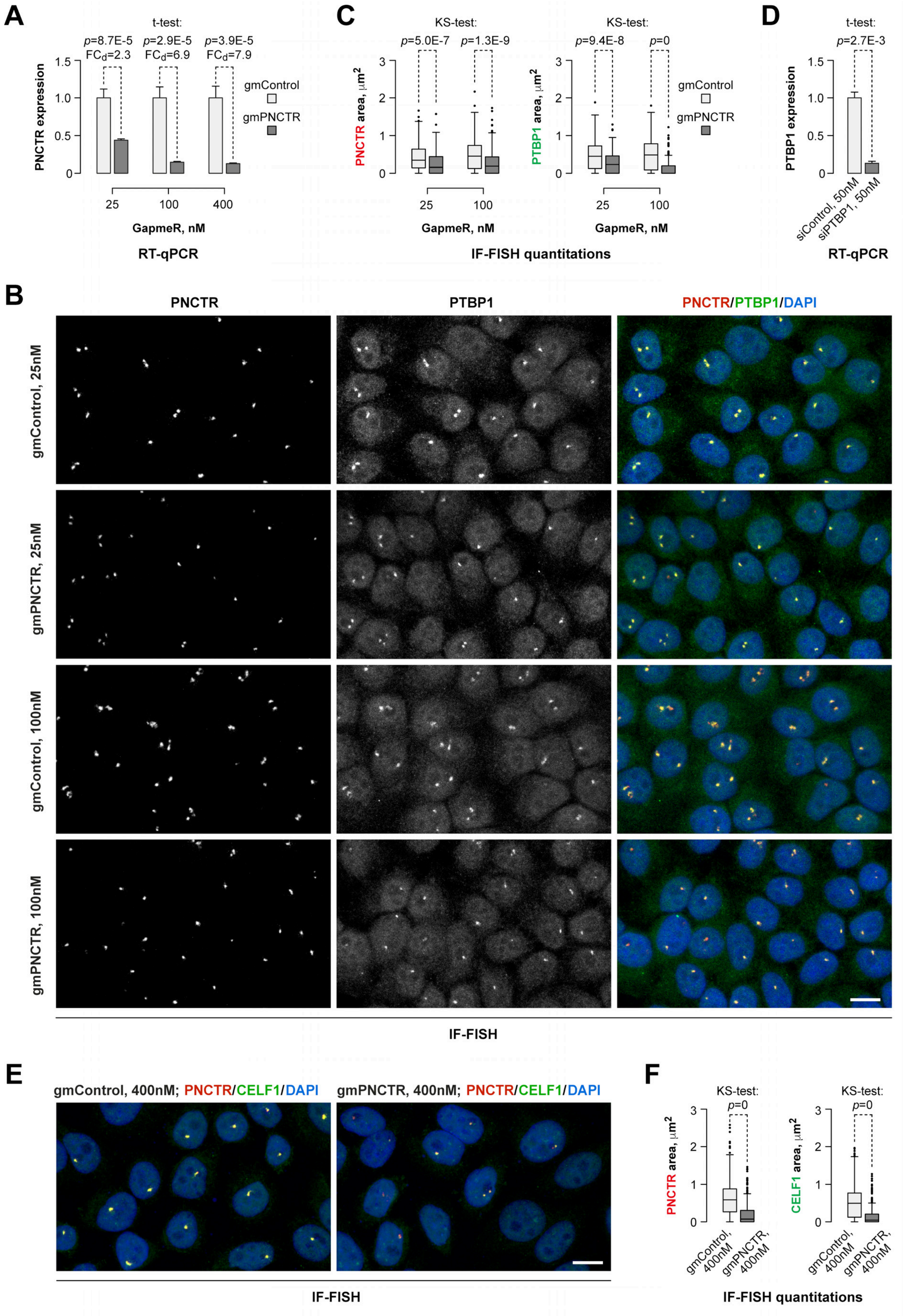

Figure S4. Effect of PNCTR knockdown on the PNC, Related to Figure 3.

(**A**) HeLa cells were treated with 25, 100 or 400 nM of gmControl or gmPNCTR for 24 hours and the effect of these treatments on PNCTR expression was analyzed by RT-qPCR with RqP1 primers. Note that although all three concentrations of gmPNCTR dampen PNCTR levels compared to siControl, the down-regulation effect (fold downregulation, FC_d) is the strongest at 400 nM. Data are averaged from 3 independent transfection experiments ±SD and compared by a two-tailed t-test assuming unequal variances.

(**B**) IF-FISH analyses of HeLa cells carried out as in Fig. 3A but using lower gapmer concentrations (25 and 100 nM). Note that gmPNCTR diminishes the size of the PNCTR and PTBP1 dots but this effect is less pronounced compared to 400 nM gmPNCTR used in Fig. 3A.

(**C**) Changes in the PNCTR and the PTBP1 dot areas per individual nuclei in (B) analyzed by a two-sided Kolmogorov-Smirnov (KS) test.

(**D**) Efficacy of PTBP1 knockdown by siPTBP1 in the experiment described in Fig. 3D-F was analyzed by RT-qPCR. Data are averaged from 3 experiments ±SD and compared by a two-tailed t-test assuming unequal variances.

(**E**) HeLa cells were treated for 24 hours with 400 nM gmControl or gmPNCTR and co-stained with a CELF1-specific antibody and a PNCTR-specific FISH probe. Note that gmPNCTR dampens PNC-localized signals in both the PNCTR and CELF1 channels suggesting that PNCTR plays a critical role in the PNC assembly. Scale bars in (B, E), 10 μm.

(**F**) The above conclusion is confirmed by comparing gmPNCTR-induced changes in the PNCTR and the CELF1 dot areas by a two-sided KS test.

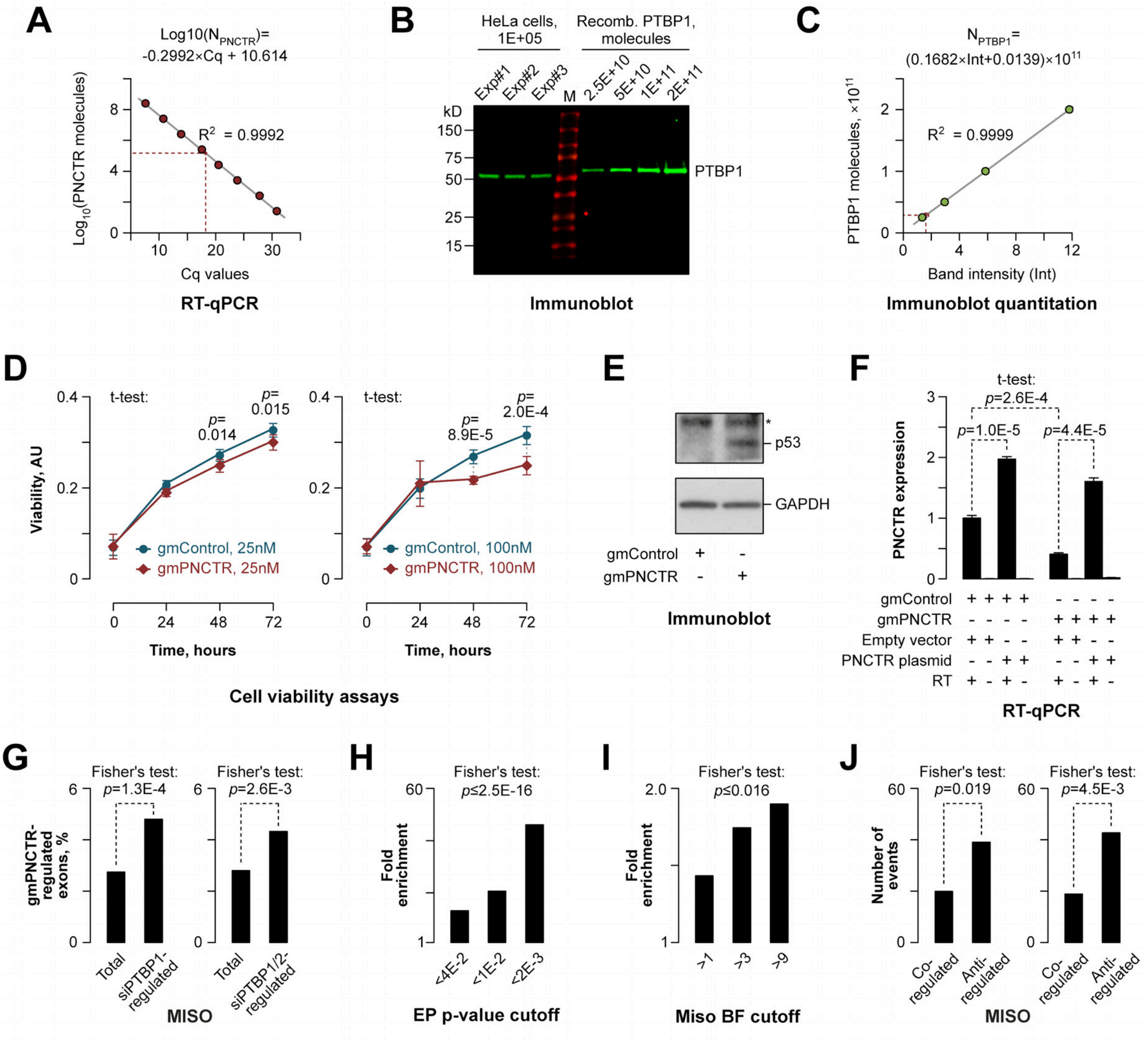

Figure S5. Quantitation of PNCTR abundance and elucidating its biological and molecular functions in HeLa cells, Related to Figures 4, 5 and 6.

(**A**) Absolute RT-qPCR analysis of PNCTR abundance in HeLa cells. The dashed line shows RT-qPCR signal from 4,000 HeLa cells on a linear regression line plotted for known amounts of a synthetic PNCTR RNA fragment.

(**B**) PTBP1 expression levels were estimated by comparing immunoblot signals derived from 100,000 HeLa cells (three lanes on the left) with recombinant PTBP1 band intensities (four lanes on the right). Protein marker positions are indicated on the left.

(**C**) Linear regression line plotted for recombinant PTBP1 samples in (E). The dashed line marks PTBP1 signal corresponding to 100,000 HeLa cells. Panels (A) and (C) also show the equations used to calculate PTBP1 and PNCTR abundance as well as the R^2 values for the calibration curve. These analyses estimate PNCTR abundance at ~36 and PTBP1 abundance at ~286,000 molecules per typical HeLa cell.

(**D**) Time courses of HeLa cell viability assayed as in Fig. 5C but using lower gapmer concentrations (25 and 100 nM). Note that the gmPNCTR-treated cultures begin to lag behind the gmControl-treated ones from 24 h.p.t. Data are averaged from 6 transfection experiments ±SD and compared by a two-tailed t-test assuming unequal variances.

(**E**) Immunoblot analysis showing detectable up-regulation of the pro-apoptotic tumor suppressor protein p53 in HeLa cells treated for 24 hours with 400 nM of gmPNCTR but not gmControl. The asterisk marks an unspecific band recognized by the p53-specific antibody. GAPDH is a lane loading control.

(**F**) RT-qPCR analysis of PNCTR expression changes in HeLa cells in the experiment described in Fig. 5G-H. As expected, no signal is detected in RT-negative controls.

(**G**) Fisher's exact tests showing that gmPNCTR-regulated alternative splicing events are significantly enriched among those regulated by knockdown of (*left*) PTBP1 (siPTBP1) or (*right*) both PTBP1 and PTBP2 (siPTBP1/2), as compared to the entire list of alternative splicing events examined by MISO (Total).

(**H-I**) Fold enrichment of the gmPNCTR events among the siPTBP1-regulated ones increases with an increase in shortlisting stringency, i.e. when using (H) smaller ExpressionPlot p-value or (I) larger MISO Bayes factor (BF) cutoffs.

(**J**) Fisher's exact tests demonstrating that MISO-identified alternative events controlled by both (*left*) gmPNCTR and siPTBP1 or (*right*) gmPNCTR and

siPTBP1/2 are more frequently regulated in opposite directions (anti-regulated) than the same direction (co-regulated).

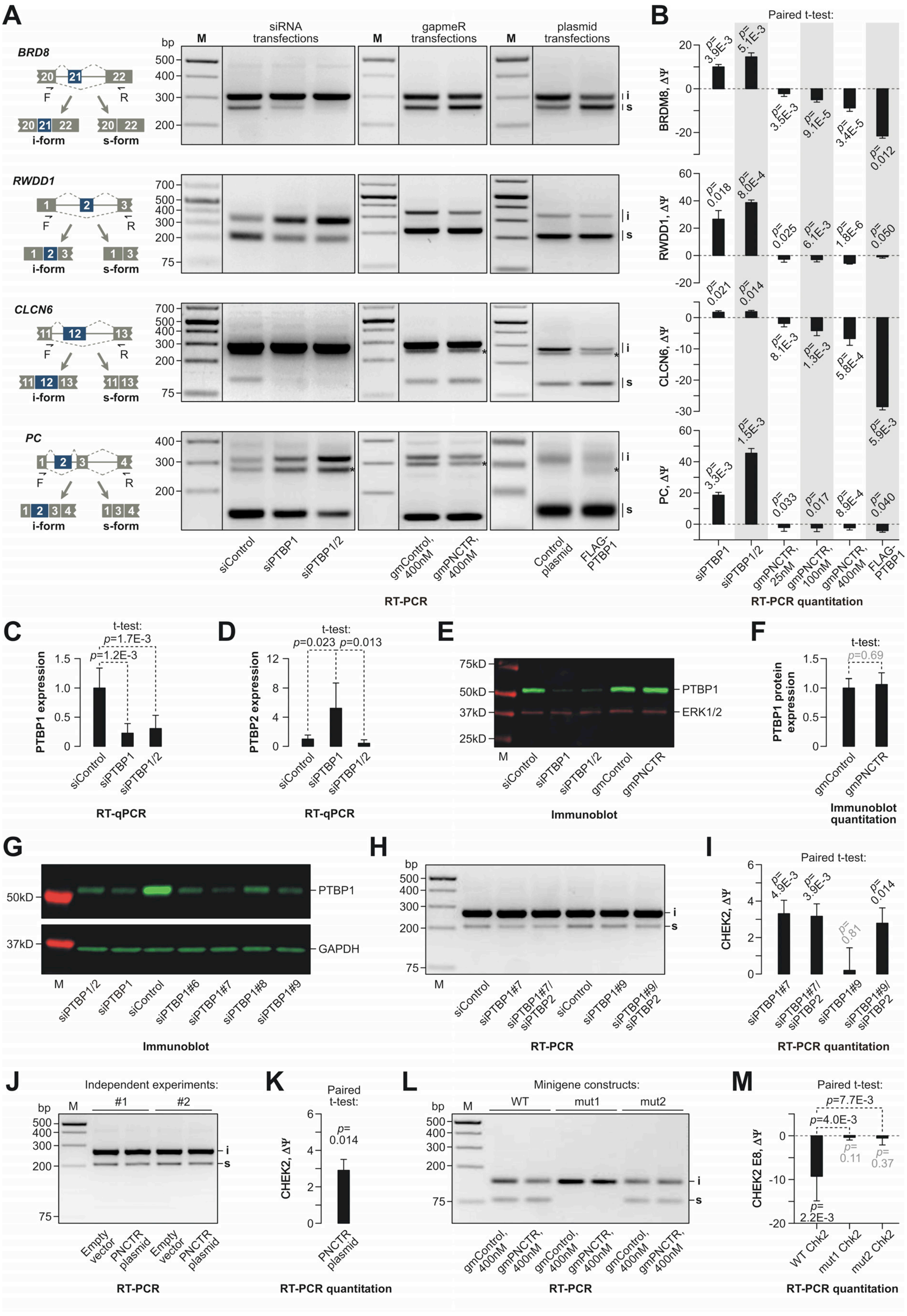
A
BRD8
RWDD1
CLCN6
PC
F
R
i-form
s-form
bp
M
siRNA transfections
gapmeR transfections
plasmid transfections
i
s
siControl
siPTBP1
siPTBP1/2
gmControl, 400nM
gmPNCTR, 400nM
Control plasmid
FLAG-PTBP1
RT-PCR
B
Paired t-test:
BRDM8, ΔΨ
RWDD1, ΔΨ
CLCN6, ΔΨ
PC, ΔΨ
gmPNCTR, 25nM
gmPNCTR, 100nM
gmPNCTR, 400nM
RT-PCR quantitation
C
t-test:
p=1.7E-3
p=1.2E-3
PTBP1 expression
RT-qPCR
D
p=0.023 p=0.013
PTBP2 expression
E
PTBP1
ERK1/2
gmControl
gmPNCTR
Immunoblot
F
p=0.69
PTBP1 protein expression
Immunoblot quantitation
G
PTBP1
GAPDH
siPTBP1#6
siPTBP1#7
siPTBP1#8
siPTBP1#9
H
siPTBP1#7/ siPTBP2
siPTBP1#9/ siPTBP2
I
CHEK2, ΔΨ
J
Independent experiments:
#1
#2
Empty vector
PNCTR plasmid
K
Paired t-test:
p= 0.014
L
Minigene constructs:
WT
mut1
mut2
M
p=7.7E-3
p=4.0E-3
p= 2.2E-3
p= 0.11
p= 0.37
CHEK2 E8, ΔΨ
WT Chk2
mut1 Chk2
mut2 Chk2

Figure S6. PNCTR controls splicing of several pre-mRNAs by antagonizing PTBP1 function, Related to Figure 6.

(**A**) Alternative splicing changes in transcripts regulated by PNCTR, PTBP1 and possibly PTBP2 were analyzed by RT-PCR. *Left*, diagrams of the four alternative events additionally showing RT-PCR primes (F and R) used to analyze their splicing status. *Right*, RT-PCR analyses of HeLa cells demonstrating that knocking down PTBP1 individually (siPTBP1; 50 nM) or in combination with PTBP2 (siPTBP1/2; 25 nM each) stimulates inclusion of the four cassette exons while PNCTR knockdown (gmPNCTR, 400 nM) promotes their skipping, as compared to the corresponding controls: siControl (50 nM) and gmControl (400 nM), respectively. Similar to the effect induced by PNCTR knockdown, PTBP1 over-expression in cells transiently transfected with a FLAG-PTBP1-encoding plasmid promotes exon skipping Unspecific RT-PCR products are marked by asterisks.

(**B**) Quantification of the effects in (A) presented as differences in percent-spliced-in values [$\Delta\Psi$; (Wang et al., 2008)] between experimental treatments and the corresponding controls. Positive $\Delta\Psi$ values indicate an increase and negative, a decrease in cassette exon inclusion. Also shown are similar quantifications done for HeLa cells transfected with lower gapmer concentrations (25 and 100 nM). All data are averaged from 3 experimentally independent comparisons ±SD and analyzed by a paired t-test.

(**C**) RT-qPCRs confirming efficiency of the PTBP1 mRNA knockdown in HeLa cells by siPTBP1 and siPTBP1/2 compared to siControl.

(**D**) RT-qPCRs showing that expression of the PTBP2 mRNA increases in response to siPTBP1 treatment as a result of previously described nonsense-mediated decay-dependent mechanism (Boutz et al., 2007; Makeyev et al., 2007; Spellman et al.,

2007). As expected, this increase is cancelled out by treating HeLa cells with a combination of siPTBP1 and siPTBP2 (siPTBP1/2). Data in (C-D) are averaged from 3 experiments ±SD and compared by a two-tailed t-test assuming unequal variances.

(**E**) Immunoblot analysis showing that siPTBP1 and siPTBP1/2 dampen PTBP1 protein expression in HeLa cells as compared to siControl. On the other hand, no difference in PTBP1 levels is detected in HeLa cells treated with gmControl and gmPNCTR. ERK1/2-specific antibody was used to confirm that the lanes were loaded equally.

(**F**) Quantification of PTBP1 protein expression in gmControl and gmPNCTR treated HeLa cells averaged from 3 independent experiments ±SD and compared a by a two-tailed t-test assuming unequal variances. No significant difference is detected.

(**G-I**) High-efficiency knockdown of PTBP1 is sufficient to reduce CHEK2 exon 8 skipping. (G) Immunoblot analysis of HeLa cells transfected with 4 individual PTBP1-specific siRNAs (siPTBP1#6, siPTBP1#7, siPTBP1#8, and siPTBP1#9), an equimolar mixture of these four siRNAs used throughout our study (siPTBP1) or both siPTBP1 and siPTBP2 (siPTBP1/2). Note that siPTBP1#7 is the most potent of the 4 siRNAs followed by siPTBP1#9, siPTBP1#6 and siPTBP1#8. (H) RT-PCR assay showing that siPTBP1#7 reduces skipping of exon 8 in CHEK2 mRNA both with and without siPTBP2. On the other hand, siPTBP1#9 performing similarly to the siPTBP1 mixture leads to a detectable splicing change only in combination with siPTBP2. (I) Quantification of the difference in exon 8 inclusion between the experimental and control (siControl) samples in (H) averaged from 3 independent comparisons ±SD and analyzed by a paired t-test.

(**J-K**) Transient overexpression of a (UC)n-containing PNCTR fragment in HeLa cells leads to a modest but statistically significant reduction in skipping of CHEK2

exon 8. (J) RT-PCR assay of two independent transfection experiments. (K) Quantification of the difference in exon 8 inclusion between the PNCTR-plasmid and empty-vector samples (J) averaged from 3 experiments ±SD and analyzed by a paired t-test.

(**L-M**) To address functional significance of the PTBP1 binding sites preceding CHEK2 exon 8, we prepared three minigene constructs where exon 8 in its immediate intronic context was inserted into a recombinant constitutively spliced intron. One of these constructs (WT) had a natural exon 8 splicing acceptor/polypyrimidine tract with seven PTBP1-specific YUCUYY and YYUCUY motifs. In the other two minigenes, this sequence was substituted with splicing acceptor/polypyrimidine tract units from the adenovirus major late pre-mRNA (mut1) or CHEK2 exon 6 (mut2), both lacking the YUCUYY and YYUCUY motifs. HeLa cells pre-transfected with these constructs for 5 hours were treated with either gmControl or gmPNCTR for another 24 hours and the inclusion of exon 8 into minigene transcripts was analyzed by RT-PCR using Minigene_F and Minigene_R primers (Table S5). (L) RT-PCR shows that, PNCTR knockdown promotes a detectable increase in skipping of exon 8 in the WT minigene, similar to the effect of this treatment on the endogenous CHEK2 pre-mRNA. On the other hand, the two mutant minigenes do not show increased skipping of exon 8 in response to gmPNCTR. (M) Quantification of the data in (L) averaged from 6 independent comparisons ±SD and analyzed by a paired t-test.

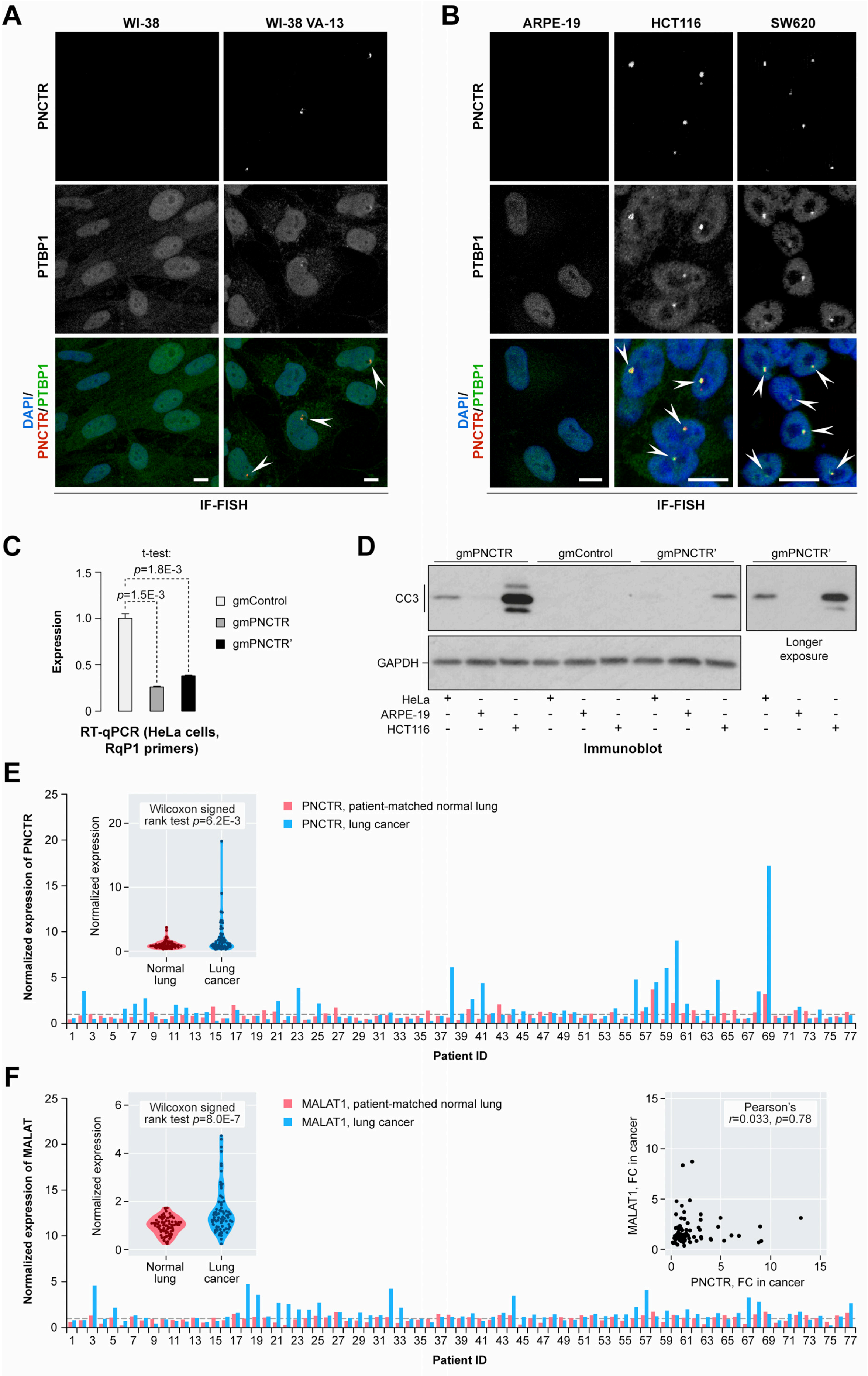

A
WI-38
WI-38 VA-13
PNCTR
PTBP1
DAPI/PNCTR/PTBP1
IF-FISH
B
ARPE-19
HCT116
SW620
PNCTR
PTBP1
DAPI/PNCTR/PTBP1
IF-FISH
C
t-test:
p=1.8E-3
p=1.5E-3
Expression
gmControl
gmPNCTR
gmPNCTR'
RT-qPCR (HeLa cells, RqP1 primers)
D
gmPNCTR
gmControl
gmPNCTR'
gmPNCTR'
CC3
GAPDH
Longer exposure
HeLa
ARPE-19
HCT116
Immunoblot
E
Normalized expression of PNCTR
Wilcoxon signed rank test p=6.2E-3
Normalized expression
Normal lung
Lung cancer
PNCTR, patient-matched normal lung
PNCTR, lung cancer
Patient ID
F
Normalized expression of MALAT
Wilcoxon signed rank test p=8.0E-7
Normalized expression
Normal lung
Lung cancer
MALAT1, patient-matched normal lung
MALAT1, lung cancer
Pearson's r=0.033, p=0.78
MALAT1, FC in cancer
PNCTR, FC in cancer
Patient ID

Figure S7. PNCTR is up-regulated in a wide range of cancer cells, Related to Figure 7.

(**A**) PNC-localized PNCTR and PTBP1 signals are detectable in the transformed WI-38 VA-13 cell line but not in its non-transformed parent, WI-38.

(**B**) PNCTR signal also co-localizes with PTBP1 in the PNC (arrowheads) in colon cancer lines HCT116 and SW620. Conversely, no PNC is detected in the non-transformed epithelial cell line ARPE-19. Scale bars in (A-B), 10 μm.

(**C-D**) PNCTR knockdown is an efficient trigger of an apoptotic program in carcinoma cell lines but not in normal epithelial cells. (C) RT-qPCR analysis of PNCTR knockdown by gmPNCTR and another PNCTR-specific gapmer, gmPNCTR'. (D) Two carcinoma cell lines (HeLa and HCT116) and a non-transformed epithelial cell line (ARPE19) were transfected for 24 hours with 400 nM of either control (gmControl) or PNCTR-specific gapmers (gmPNCTR and gmPNCTR') and analyzed by immunoblotting with CC3-specific antibodies. Note that both PNCTR-specific gapmers induce detectable expression of CC3 in HeLa and HCT116 but not ARPE19, although the effect of gmPNCTR' is somewhat less potent than that of gmPNCTR. Stronger induction of CC3 in HCT116 compared to HeLa might be due to the presence of fully functional pro-apoptotic tumor suppressor p53 in the former but not the latter cell line.

(**E-F**) Publicly available RNA-seq data for 77 patient-matched pairs of lung cancer and normal lung biopsies (Ju et al., 2012) were analyzed with Kallisto (Bray et al., 2016) to determine the expression of (E) PNCTR and (F) the nuclear speckle-associated lncRNA MALAT1 originally identified in metastatic lung cancer samples (Sun et al., 2017). In both cases, TPM expression values were normalized to the normal lung average (dashed lines). The insets on the left in (E-F) show that both

lncRNAs are significantly up-regulated in cancer, based on pairwise comparisons by Wilcoxon signed rank test. The inset on the right in (F) demonstrates that cancer-specific changes in PNCTR and MALAT1 abundance do not correlate (Pearson's correlation coefficient r=0.033) suggesting that different mechanisms may control expression of the two lncRNAs.
